# Benchmarks for low back pain in general practice in Flanders: electronic audit of INTEGO

**DOI:** 10.1186/s12875-024-02644-6

**Published:** 2024-12-20

**Authors:** Rico Paridaens, Bert Vaes, Steve Van den Bulck, Justine Soetaert

**Affiliations:** 1 Ghent University, Ghent, Belgium; 2 KU Leuven, Leuven, Belgium; 3 Research Group Healthcare and Ethics, UHasselt, Hasselt, Belgium

**Keywords:** Low back pain, Quality of care, Quality indicator, Family medicine, Electronic health records

## Abstract

**Background:**

Low back pain (LBP) is one of the most frequent reasons for encounter in general practice. Yet results from literature show adherence to clinical practice guidelines is low. Audit & feedback is a well-known strategy to improve adherence to guidelines. Benchmarking is an important step in the audit & feedback process. The objective of this study was to develop data-derived benchmarks for low back pain quality indicators.

**Methods:**

Four electronic health record extractable quality indicators were selected from an existing indicator set developed by CEBAM, an independent, multidisciplinary and interuniversity medical scientific institute in Belgium. Data from 2021-2022 from INTEGO, a general practice morbidity registry, were used to calculate benchmarks for the four quality indicators. The Achievable Benchmark of Care methodology was used to create benchmarks based on the performance of the 10% best-performing practices.

**Results:**

The following benchmarks were derived: 4.2% prescription for medical imaging, 12.7% prescription for opioids, 27.2% for prescription for non-steroidal anti-inflammatory drugs or acetaminophen, 37.7% prescription for physical therapy and 11.9% prescription for work absenteeism.

**Conclusions:**

Benchmarks for four electronic health record-extractable quality indicators have been established. They can be used for an electronic audit & feedback tool in primary practice in Flanders or other quality improvement initiatives.

**Supplementary Information:**

The online version contains supplementary material available at 10.1186/s12875-024-02644-6.

## Background

Low back pain (LBP) is one of the most frequent reasons for encounter in general practice. The mean prevalence is estimated to be 18.3%, and one-month prevalence 30.8% [1]. It’s also the leading cause of years lived with disability worldwide (7.4% of years lived with disability) [2]. LBP can be caused by a specific pathology such as infection, tumour, osteoporosis, lumbar spine fracture, structural deformity, inflammatory disorder, radicular syndrome, or cauda equina syndrome; but most cases of LBP are non-specific low back pain (NSLBP) [3–5]. NSLBP is in most cases a self-limiting disease and most patients improve regardless of treatment [6]. Clinical practice guidelines recommend avoiding routine imaging and other diagnostic tests;, and recommend nonpharmacological treatment as the first choice [4, 6, 7]. Yet one quarter of patients who present with LBP in primary care are referred for imaging [8], and many patients receive a prescription for analgesics (48% in the UK and 61% in Australia) [9–11]. This shows that there is still a long way to go before these clinical practice guidelines will be achieved in actual practice.

Unnecessary imaging for low-back pain has been associated with poorer patient outcomes, increased radiation exposure and higher health care costs and may be accounted for 7% of direct costs associated with low-back pain [12, 13]. In the UK back pain is also one of the commonest reasons for prescribing a sickness certificate [14]. Prescribing physical therapy may lower utilization of high cost medical services (such as imaging and emergency care visits) as well as lower opioid use [15]. Last, non-opioid analgesics (NSAIDs or acetaminophen) have a statistically significant pain reducing effect over opioids. Opioids may potentially have an effect in acute setting for low-back pain, but the risk of harm (adverse effects and dependence) greatly outweighs the benefits. A American study in 2001 reported that opioid prescriptions for low back pain may account for up to 48% of expenditures for all prescribed drugs for low back pain [9, 11, 16–19].

Audit and feedback (A&F) is frequently used as a strategy to improve professional practice. A Cochrane systematic review defined A&F as “summary of clinical performance over a specified period of time” [20]. Systematic reviews of the topic showed that it can effectively improve quality of care [20, 21]. A&F is most effective when baseline performance is low, when provided by a supervisor and when it is provided continuously [20]. Manual A&F however can be time-consuming and costly. The evolution in electronic health records (EHRs) with EHR-extractable quality indicators allows for this auditing process to be automated and implemented on large scale. This can make electronic A&F (eA&F) a cost-effective alternative to manual A&F [22–24]. A similar initiative has already been developed for diabetes care in Belgium where each practice receives an automated report on a regular base for some EHR-extractable quality indicators for diabetes care (the “diabetes barometer”) [25].

From a methodological perspective, A&F consists of a “quality loop”: a topic is chosen for which a set of criteria and targets is defined, current clinical practice is evaluated, especially in terms of process or outcome, and suggestions for improvement are developed and applied [26]. One of the first steps (defining a set of criteria and targets) is often done by defining quality indicators. Quality indicators are are measurable items referring to structures, processes, and outcomes of care. They should be defined in a well-structured and transparent way to produce high quality standards since they may have far-reaching consequences (for example in pay-for-performance models) [27]. Afterwards the process of “benchmarking” is used to set a targets for quality improvement. Webster’s Dictionary defines a benchmark as “something that serves as a standard by which others can be measured”. It’s an important step of the A&F process as it provides as it sets goals for each quality indicator and thus a reference for feedback and comparison [28]. The Achievable Benchmarks of Care (ABC™) approach is a benchmarking method that sets “real world” goals. It defines “top performance” using the “pared mean”, defined as the average performance of the subset of those providers with the highest scores for the indicator under consideration. The subset includes the top-ranked providers down to the point whereby at least 10% of the patient pool across all providers is selected [29]. The use of achievable benchmarks has been shown to be effective [28, 30, 31].

The aim was to develop data-derived benchmarks for low back pain quality indicators that can be used in an electronic A&F tool for low back pain in primary care practice in Flanders. The benchmarks may also be used for reporting and quality improvement in patients with low back pain in other countries since benchmarks, and especially achievable benchmarks are an important tool for quality improvement.

## Methods

### Data source

INTEGO, a general practice morbidity registry in Flanders (Belgium), consists of a large pool of patient data, that are systematically collected from electronic healthcare systems of general practitioners and corresponds to more than 6.2% of the Flemish population in 2022. The data were pseudonymized and followed national privacy legislation [32]. The overall project was approved by the Research Ethics Committee UZ/KU Leuven (MP018709). Patient characteristics and individual consultation data were collected. Some data is further encoded such as diagnosis in International Classification of Primary Care-2nd edition (ICPC-2) [33], medication in Anatomical Therapeutic Chemical Classification (ATC) code [34], specifics about prescriptions for medical imaging, absence and physical therapy [32].

### Data sample

A cross-sectional study design was used. Patients aged eighteen years or older with a global medical record (GMR) in the practice and with LBP were identified. LBP was defined as at least one consultation with the ICPC-2 diagnostic codes: L02, L03, L84 or L86. Data for 2021 and 2022 were extracted from INTEGO. Only practices that contributed more than ten cases of LBP were included in the analysis. Practices with a zero value for two or more indicators were excluded for all indicators because this may indicate the data is not valid.

### Ethics approval and consent to participate

Ethical approval was granted by the Research Ethics Committee UZ/KU Leuven (MP018709, Supplementary Material 2 and 3). All data extracted from electronic medical records of general practitioners were stored pseudonymized in the INTEGO database with strict security regulations under supervision of Healthstat, and followed national privacy legislation. INTEGO is approved by the Informatie Veligheidscomité in Belgium (IVC/KSZG/23/424, Supplementary Material 1). Patient specific data was analysed inside the Healthstat environment by the main researcher (Rico Paridaens) and only anonymised practice data was extracted for further analysis on a local computer using SPSS. There was no patient or public involvement in this study.

### Low back pain quality indicators

The Belgian Centre for Evidence-Based Medicine (CEBAM), an independent, multidisciplinary and interuniversity medical scientific institute [35] and Federaal Kenniscentrum voor de Gezondheidszorg (KCE) defined a list of quality indicators for low back pain using a modified Delphi method and based upon existing indicator sets (National Institute for Health and Care Excellence [NICE], Institut für Qualitätssicherung und Transparenz im Gesundheitswesen [IQTIG], Nederlands Huisartsen Genootschap (NHG), Healthcare Effectiveness Data and Information Set [HEDIS] and International Consortium for Health Outcomes Measurement [ICHOM] guidelines) [36]. Based upon these quality indicators a list was made of four quality indicators that can be evaluated in HER (Table 1). For the second quality indicator “Proportion of adults with low back pain and a prescription for opioids versus adults with low back pain with a prescription for non-steroidal anti-inflammatory drugs or acetaminophen” we slightly had to alter the definition for practical reasons. The original definition allowed for zero-values in the denominator which can result in calculation problems. So, we divided it in 2 quality indicators: A. “Proportion of adults with low back pain with a prescription for non-steroidal anti-inflammatory drugs or acetaminophen”; B. “Proportion of adults with low back pain with a prescription for opioids”.

**Table 1:** Electronic medical record extractable quality of care indicators for low back pain

**Indicator**	**Type of indicator**
1. Proportion of adults with low back pain where imaging is requested	process
2. **Original definition**: Proportion of adults with low back pain and a prescription for opioids versus adults with low back pain with a prescription for non-steroidal anti-inflammatory drugs or acetaminophen **Altered definition**: A. Proportion of adults with low back pain with a prescription for non-steroidal anti-inflammatory drugs or acetaminophen B. Proportion of adults with low back pain with a prescription for opioids	process
3. Proportion of adults with low back pain with a physical therapy prescription or with “back school” prescriptions	process
4. Proportion of adults with low back pain on medical leave	process

Quality of care indicators for low back pain that are extractable from electronic medical record in Flanders. Filtered from the initial set of indicators as defined by CEBAM in 2021 using a modified Deplhi method using existing indicator sets [36]

### Statistical analysis

**Fig. 1:**
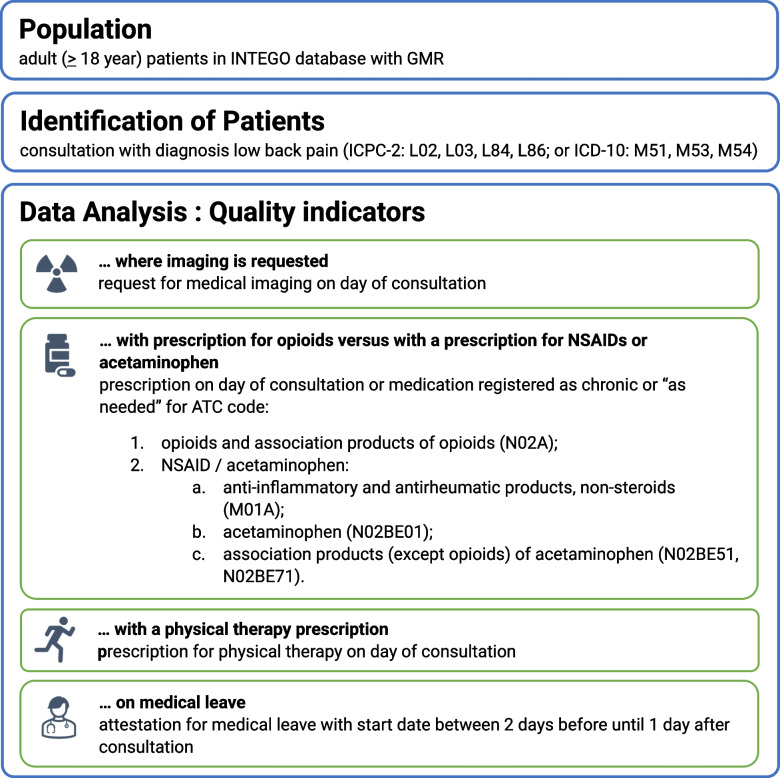
Summary of the R script developed for the INTEGO database. Summary of the R script developed for the INTEGO database with specifics about how the quality indicators were calculated. NSAIDs: non-steroidal anti-inflammatory drugs; ICPC-2: International Classification of Primary Care—2nd edition; ATC: Anatomical Therapeutic Chemical Classification

A script was developed in the programming language R to automatize the data analysis process. The INTEGO database was filtered to select consultations for LBP. Each consultation received a PASS or FAIL score for each of the quality indicators. Figure 1 shows more in detail how the INTEGO database was filtered, and which criteria were used to evaluate each of the quality indicators. Consultations were then grouped per patient because it was impossible to evaluate whether different consultations were part of the same episode of LBP. If patients had multiple consultations for LBP the worst score for each quality indicator was used (except for quality indicator 3 where the best score was used). The data were pooled for each practice and a score for each quality indicator per practice was calculated by counting the number of patients that had a PASS value divided by the total number of patients with the diagnosis LBP in the practice.

The data was then further analysed in IBM SPSS Statistics (Version 29). The normality of the distribution was evaluated using the Kolmogorov-Smirnov test, QQ-plot, skewness and histogram. The ABC™ methodology was used to calculate benchmarks for the four quality indicators. The overall indicator performance was assessed using Kiefe et al.’s algorithm to determine the minimum sufficient denominator (MSD) (i.e. eligible patients). If needed a Bayesian adjustment was used to calculate the practice’s adjusted performance fraction [28, 29, 37]. The benchmark was calculated as the mean of the practices in the 10th upper percentile of performance. This was used as an alternative to Weissman’s original definition of the ABC™ benchmark because it is easier to implement for continuous feedback and because the benchmark did not significantly differ from the original method of calculation (*p* > 0.05 for Independent Samples Student t-test and Levene's Test for Equality of Variances). For each of the quality indicators is reported (1) the mean, standard deviation (SD) and 95% confidence interval (95% CIs), (2) the ABC™ benchmark and the amount of practices at or above the benchmark, (3) descriptive statistics (N, median, range, 10th, 25th, 75th and 90th percentile of the practices included in the benchmark calculation and (4) the practices in the audit with patient samples below the MSD.

## Results

The INTEGO database included 132 practices for 2021 and 2022. Nine practices were excluded from further analysis because of incomplete data in the INTEGO database (ex. no coded diagnosis for LBP, incomplete consultation data…). Any practice with less than 10 patients with LBP or with two or more indicators without data (zero-values) was excluded (15 practices). Table 2 describes the characteristics and demographics of the patients and practices included in the INTEGO low back pain audit from 2021–2022. The total GMR population was 318,744 GMR patients for the 108 practices. The prevalence of LBP was 144.1 patients / 1,000 GMR patients (*n* = 45,938). There was a slight overweight of female patients (54.5%).

**Table 2:** Characteristics of patients and practices

**Characteristic**	**N (%)**
**Patients consulting with LBP**	45,938 (144.12^a^)
**Patient sex**	
**Male**	20,923 (45.5)
**Female**	25,011 (54.5)
**Unknown**	4 (0.0)
**Patient age**	
**18-24**	2,452 (5.3)
**25-34**	6,574 (14.3)
**35-44**	8,585 (18.7)
**45-54**	8,470 (18.4)
**55-64**	8,562 (18.6)
**65-74**	5,737 (12.5)
**> 75**	5,558 (12.1)
**Urbanisation practices**	
**Urban**	87.2 %
**Rural**	12.8 %

Characteristics of practices included (*N* = 108) and patients (*N* = 318,744) in INTEGO low back pain audit 2021-2022. Nine practices were excluded because of incomplete data. 15 practices were excluded because they had less than 10 patients with LBP or because they had no patients for 2 or more benchmark scores. Demographic information of the practices in INTEGO on 01/01/2023 divided in urbanisation as defined by the European Commission [38] based on the Spatial Plan for Flanders [39]

*LBP* low back pain

^a^Prevalence (/ 1,000 GMR patients)

Table 3 summarizes the overall performance for each quality indicator and the target benchmarks using the ABC™ methodology. There was a wide range of performances for all four quality indicators ranging from 45.9% for prescription for opioids to 16.0% for prescription for imaging. Using the MSD a Bayesian adjustment was applied to the performance factor for three practices for prescription for imaging and for two practices for prescription for physical therapy. The defined benchmarks using the ABC™ methodology ordered from highest target to lowest are 4.2% for prescription for medical imaging, 11.9% for prescription for medical leave, 12.7% for prescription for opioids, 27.2% for prescription for NSAIDs and 37.7% for prescription for physical therapy. Only approximately 5 to 10% of all the practices reached the ABC™ benchmark, except for indicator 2.B prescription for opioids, where 17.6% of practices reached the ABC™ benchmark.

**Table 3:** Achievable benchmark of care in INTEGO low back pain audit 2021-2022

**Low back pain quality indicator**	**Overall performance**			**Benchmark**	
	**Mean (%)**	**SD (%)**	**95% CI (%)**	**ABC™ (%)**	**Number of practices at or above the ABC™ benchmark**
**1. Imaging**	15.97	0.73	14.51-17.42	4.20	6
**2.A NSAIDs or acetaminophen**	45.93	1.00	44.03-47.84	27.16	3
**2.B Opioids**	18.92	0.75	17.49-20.34	12.74	19
**3. Physical therapy**	19.47	1.08	16.32-21.63	37.67	6
**4. Medical leave**	23.16	0.66	21.85-24.47	11.87	7

The ABC™ benchmark was calculated as the 10% best performing practices (*N* = 11). For each benchmark the amount of practices that reached the benchmark is reported of the total of 108 practices. The overall performance of the whole population is also reported as a mean and 95% confidence interval

*LBP* low back pain, *NSAIDs* non-steroidal anti-inflammatory drugs, *95% CI* 95% confidence interval, *ABC™* Achievable Benchmark of Care, *SD* Standard deviation

^a^Eligible patients is the total number of patients with LBP receiving NSAIDs, acetaminophen or opioids

The number of patients included in the benchmark calculation (eligible patients) per practice varies strongly (range 13 to 780 patients). Only for prescriptions for physical therapy some bigger practices were included (range 44-1542) (Table 4).

**Table 4:** Distribution of eligible patient volume per practice in INTEGO low back pain audit 2021–2022

**Descriptive**	**1. Imaging**	**2.A NSAIDs or acetaminophen**	**2.B Opioids**	**3. Physical therapy**	**4. Medical leave**
N	1,991	3450	2539	5,933	1,980
Range	23-455	23-780	27-692	44-1,542	16-464
10th percentile	23.4	23.8	27.0	56.2	18.2
25th percentile	27.0	105.0	44.0	178.0	44.0
Median	86.0	358.0	138.0	393.0	193.0
75th percentile	393.0	448.0	425.0	679.0	228.0
90th percentile	446.0	725.0	654.0	1532.4	451.0

Descriptive statistics of the Eligible Patient Volume. This is the volume of patients included in the ABC™ benchmark calculation

## Discussion

This study provides benchmarks using the ABC™ methodology for four low back pain quality indicators that can be measured in the EHR in Flanders. Adjustments were made for practices with small numbers of eligible patients. To allow replication of our work we included detailed indicator definitions. To assess precision of the benchmarks we reported the median and range of eligible patients within the practices included. These benchmarks are derived from a representative sample of patients presenting with low back pain in primary care in 108 general practices in Flanders representing a GMR population of more than 300,000 patients.

The four quality indicators we defined are key-messages in most guidelines [4–7, 40]. The results on overall performance for these quality indicators in literature varies strongly because of difference in definition and method of analysis used [8, 15, 16, 41–48]. This makes comparison to previous literature difficult. To our knowledge this study is the first to define benchmarks for LBP. We defined our benchmarks using existing indicator sets in other countries so it can be used in other countries to reproduce and compare results. This could allow for a more international approach in the treatment for LBP and more targeted interventions as it would help in evaluating region-based trends and evolutions in treatment of LBP.

Unfortunately, we could not extract data for 5 other quality indicators that were selected by CEBAM because currently they are not extractable in the available Belgian EHRs for primary care. And while the INTEGO database is under constant review for validity of data, quality indicators extracted from EHRs are prone to error since they may leave error for context and not-encoded data compared to manual A&F [22–24]. As such, we had to exclude 15 practices because they failed to report data for 2 or more quality indicators indicating that it’s possible that those practices do not encode data in a standardized way. For medical imaging we could not specify the data further on the type of imaging requested since this was not encoded nor included in the data. Only information on whether or not imaging is requested is stored as encoded data. For absenteeism we could also not extract data for patients on disability benefit or patients with a special type of attestation (government employer, self-employed, etc.). In Belgium patients absent from work for more than 1 year are evaluated by the “Geneeskundige Raad voor Invaliditeit”. If they are accepted for a disability benefit they don’t need an attestation to be absent from work. We could only extract data on whether an attestation for work absence is made using the normal procedure in the EHR, however we have no encoded data on whether the patient is on a disability benefit, working or unemployed [49]. Moreover, our method also did not allow us to link a specific quality indicator to the diagnosis of low back pain but rather to the same period of consultation for low back pain. Patients could very well be receiving attestation for work, a prescription for medical imaging or physical therapy for a different medical problem than low back pain. Regarding prescriptions for medication, there is a law in Belgium that mandates encoded prescribing (except for some specific cases) [50]. Most prescriptions for medication should thus be encoded in the EHR, and these data should be least prone to error.

However, as our quality indicators are EHR-extractable in Belgium context, it allows for a cost-effective, repeatable and easy scalable way of evaluating the quality of care for LBP locally (but also regionally, nationally and internationally) [22]. Because of the size of the data sample (with only a few practices not reaching the MSD target) it also allows to calculate a representative benchmark for each quality indicator. Many other benchmarking studies struggle with a small sample size of patients [28, 29, 37]. The benchmarking target set for the quality indicators is also an achievable one and prior studies using the ABC™ method shows that it has higher improvement rates than those receiving feedback where median performance was used [51]. Another advantage of the ABC™ method is that when all practices improve and recalibration leads to higher standards, the goals will remain achievable [37]. And while A&F may be a powerful to help us improve as physicians, we must pay attention that it remains a tool to guide improvement, and not a number to achieve. Medicine remains an art where sometimes a patient tailored approach is needed. For that the achievable benchmark of care is a useful method because it does not pursue a perfect score.

In our study only approximately 5-10% of practices reached the ABC™ benchmark indicating the overall performance on the defined quality indicators is low. A Cochrane Review of A&F shows that A&F leads to small improvements in professional practice and that it’s most effective when baseline performance is low [20]. Earlier was also mentioned that low back pain remains a significant health care cost. Even only a small improvement in professional practice may have a substantial impact for any of our quality indicators, since our quality indicators are electronically extractable and can be implemented on a large scale. It can also be used using an automated script with the process of recalibration to allow for continuous feedback. The only exception is indicator 2.B regarding the prescription of opioids where almost 17.6% of the practices reached the benchmark. The average performance is in line with some results published in Australia by Michaleff et al. [43], but remarkably lower than some other results found in literature where prescriptions for opioids go up to 30-40% [16, 43, 44, 46]. This result is in line with the results published by the RIZIV (Rijksinstituut voor ziekte- en invaliditeitsverzekering). However the usage of opioids in Flanders has been increasing rapidly over the past 10 years and it remains important to strain the importance of limiting opioid use [52].

Defining benchmarks for quality indicators is yet one of the first steps in the process of A&F. Further research is needed to evaluate the effectiveness of these indicators in practice after short and long time use. Our work could be improved to define ways to evaluate episodes of LBP instead of diagnosis’s, to further specify the type of imaging and to include all patients absent from work because of low back pain. Further research is also needed to evaluate the real impact of low back pain as a cause of work absence. It may also be interesting to compare our benchmarks for these quality indicators to other countries.

## Conclusions

Our study is one of the first to define benchmarks for low back pain in general practice. The script can be used to implement an electronic audit & feedback tool in primary practice (in Flanders).

## Supplementary Information

Supplementary Material 1.

Supplementary Material 2.

Supplementary Material 3.

## Data Availability

The datasets used during the current study are available upon reasonable request to the corresponding author.

## Abbreviations

LBP: Low Back Pain
GMR: Global Medical Record
NSLBP: Non-Specific Low Back Pain
A&F: Audit and feedback
eA&F: Electronic Audit and feedback
EHRs: Electronic Health Records
ABC™: Achievable Benchmarks of Care
NSAIDs: Non-Steroidal Anti-Inflammatory Drugs
ICPC-2: International Classification of Primary Care—2nd edition
ATC: Anatomical Therapeutic Chemical Classification
NICE: National Institute for Health and Care Excellence
IQTIG: Institut für Qualitätssicherung und Transparenz im Gesundheitswesen
NHG: Nederlands Huisartsen Genootschap
HEDIS: Healthcare Effectiveness Data and Information Set
ICHOM: International Consortium for Health Outcomes Measurement
CEBAM: Belgian Centre for Evidence-Based Medicine
KCE: Federaal Kenniscentrum voor de Gezondheidszorg
MSD: Minimum Sufficient Denominator
95% CI: 95% Confidence interval
