# Supplementary material for: Benchmarks for low back pain in general practice in Flanders: electronic audit of INTEGO
Source: BMC Prim Care. 2024 Dec 20;25:431. doi: 10.1186/s12875-024-02644-6 (PMC11660715; doi:10.1186/s12875-024-02644-6)
Supplement: Supplementary file 1 — Supplementary Material 1. [file 12875_2024_2644_MOESM1_ESM.pdf]

Informatieveiligheidscomité
Kamer sociale zekerheid en gezondheid

IVC/KSZG/23/424

BERAADSLAGING NR. 13/026 VAN 19 MAART 2013, LAATST GEWIJZIGD OP 5 DECEMBER 2023, MET BETREKKING TOT DE MEDEDELING VAN GEPSEUDONIMISEERDE PERSOONSGEGEVENS DIE DE GEZONDHEID BETREFFEN, VIA HET HEALTHDATA.BE PLATFORM, IN HET KADER VAN DE SAMENSTELLING, HET GEBRUIK EN DE TER BESCHIKKING STELLING VAN EEN REGISTER VOOR EPIDEMIOLOGISCH ONDERZOEK (INTEGO)

Het Informatieveiligheidscomité, kamer sociale zekerheid en gezondheid (hierna “het Comité” genoemd),

Gelet op de verordening (EU) 2016/679 van 27 april 2016 van het Europees Parlement en de Raad betreffende de bescherming van natuurlijke personen in verband met de verwerking van persoonsgegevens en betreffende het vrije verkeer van die gegevens en tot intrekking van richtlijn 95/46/EG (Algemene Verordening Gegevensbescherming of GDPR);

Gelet op de wet van 30 juli 2018 *betreffende de bescherming van natuurlijke personen met betrekking tot de verwerking van persoonsgegevens*;

Gelet op de wet van 3 december 2017 *tot oprichting van de Gegevensbeschermingsautoriteit*, in het bijzonder artikel 114, gewijzigd bij de wet van 25 mei 2018;

Gelet op de wet van 5 september 2018 *tot oprichting van het informatieveiligheidscomité en tot wijziging van diverse wetten betreffende de uitvoering van verordening (EU) 2016/679 van 27 april 2016 van het Europees Parlement en de Raad betreffende de bescherming van natuurlijke personen in verband met de verwerking van persoonsgegevens en betreffende het vrije verkeer van die gegevens en tot intrekking van richtlijn 95/46/EG*;

Gelet op de wet van 13 december 2006 houdende bepalingen betreffende gezondheid, inzonderheid art. 42, §2, 3°;

Gelet op de machtigingsaanvraag van de KULeuven ontvangen op 17 januari 2018 en de wijzigingsaanvraag van Sciensano ontvangen op 24 maart 2020 ;

Gelet op de auditoraatsrapporten van het eHealth-platform van 12 maart 2018, 11 april 2018, 26 maart 2020 en 28 november 2023 ;

Gelet op het verslag van de heer Bart Viaene;

Beslist op 5 december 2023, na beraadslaging, als volgt:

I. VOORWERP VAN DE AANVRAAG

1. De Katholieke Universiteit van Leuven (KULeuven) legt het Comité ter goedkeuring de mededeling van gepseudonimiseerde persoonsgegevens die de gezondheid betreffen aan het Academisch Centrum voor Huisartsengeneeskunde voor in het kader een algemeen en continu morbiditeitsregistratiesysteem, het Intego-project (‘*Integrated Compurterised Network*’) genaamd. Sinds 1994 zamelt Intego morbiditeitsgegevens in die afkomstig zijn van huisartsen uit Vlaanderen. Intego verzamelt gegevens omtrent de prevalentie en incidentie van alle mogelijke ziektes en gezondheidsproblemen waarmee huisartsen geconfronteerd worden. Gebaseerd op de gegevenscollectie van Intego is het mogelijk om nieuwe onderzoekshypotheses te genereren. Intego heeft een rapporteringsfunctie op zowel nationaal als internationaal niveau om wetenschappelijke onderzoek te ondersteunen.

2. De aanvrager wenst het INTEGO-register zoals geïmplementeerd door beraadslaging nr. 13/026 van 19 februari 2013 te wijzigen. Deze machtigingsaanvraag betreft de migratie van de Intego-registratie naar healthdata.be. Meer concreet betekent dit dat de historische data gemigreerd worden naar het datawarehouse. De onderzoekers zullen rechtstreeks toegang krijgen tot het healthdata datawarehouse door hierop aan te loggen. Op die manier is er dan ook geen data-verzending vanuit healthdata.be platform naar KULeuven en zullen de onderzoekers in staat zijn om dezelfde analyses uit te voeren als voorheen. De wijzigingen aan deze beraadslaging hebben betrekking op de meegedeelde persoonsgegevens, de migratie van INTEGO naar het healthdata.be platform, de migratie van Medidoc naar CareConnect en de tussenkomst van het eHealth-platform als *Trusted Third Party*.

3. De INTEGO-gegevensbank - waarin reeds sinds 1995 gepseudonimiseerde persoonsgegevens die de gezondheid betreffen, worden geregistreerd - heeft tot doel om een steeds grotere en continue gegevensbank van ziekten te ontwikkelen, die o.a. gegevens kan verschaffen over de incidentie en de prevalentie van ziekten in Vlaanderen, over medicatie, over laboratoriumuitslagen en over achtergrondkenmerken. Op dit ogenblik worden er op basis van een jaarlijkse populatie van ongeveer 350.000 patiënten gerapporteerd, in 130 huisartsenpraktijken met 450 huisartsen. De persoonsgegevens die binnen het INTEGO-netwerk worden ingezameld, worden in gepseudonimiseerde vorm overgemaakt aan het healthdata.be platform. De gepseudonimiseerde persoonsgegevens worden geanalyseerd door onderzoekers van het Academisch Centrum voor Huisartsengeneeskunde (ACHG) van de KULeuven (verbonden aan het INTEGO-project) en worden via healthstat.be ter beschikking gesteld aan derden, met het oog op wetenschappelijk onderzoek.

4. De benodigde gepseudonimiseerde persoonsgegevens worden op automatische wijze ingezameld bij deelnemende huisartsen die gebruik maken van een bepaalde software voor het beheer van hun elektronische medische dossiers, meer bepaald CareConnect. De betrokken huisartsen ontvangen jaarlijks van de softwareproducent de vraag voor medewerking aan de registratie. Ze ontvangen een link naar een softwareprogramma dat de vereiste gegevens selecteert uit het elektronisch medisch dossier en in een bestand op de computer van de huisarts bewaard. Dit bestand wordt dan voor de verzending geëncrypteerd en doorgestuurd naar het eHealth-platform die de persoonsgegevens en de identiteit van de betrokken huisarts codeert. Patiënten worden geïnformeerd via een affiche

in de wachtzaal en de huisarts kan tijdens de consultatie nadere informatie verstrekken. De patiënt heeft de mogelijkheid om deelname te weigeren en de huisarts kan de weigering registeren in het elektronisch medisch dossier. Concreet zal er een HD4DP worden geïnstalleerd op servers van CareConnect. Datacollectie zal gebeuren door CSV-extracts vanuit de CareConnect cloud database naar HD4DP, hierbij rekening houdend met het feit dat er enkel data zullen worden geëxporteerd uit CareConnect voor de artsen die deelnemen aan Intego en voor de patiënten die zich niet verzet hebben tegen de verwerking van hun persoonsgegevens in dit register..

5. De gepseudonimiseerde persoonsgegevens die de gezondheid betreffen die in bijlage[1] worden beschreven, worden per betrokken patiënt meegedeeld. De persoonsgegevens die worden ingezameld, komen tevens uit het medisch dossier van de patiënt. Het betreft gegevens over de diagnose, onderzoeksresultaten, medische antecedenten, de behandeling van de patiënt en de beschrijving van de uitgevoerde onderzoeken en de resultaten ervan die in het kader van opvolging van de patiënt door de huisarts gecollecteerd werden.

6. Het register maakt gebruik van de architectuur zoals beschreven in *"Beraadslaging nr. 15/009 van 17 februari 2015, laatst gewijzigd op 3 maart 2020, betreffende de generieke methode voor de uitwisseling van gepseudonimiseerde en niet-gepseudonimiseerde persoonsgegevens die de gezondheid betreffen in het kader van healthdata.be en healthstat.be"*, waarmee healthdata.be de goedkeuring verkreeg om die gehele basisarchitectuur te gebruiken.

Figuur 1: Gegevensinzameling via de huisartsen met behulp van CareConnect (Corilus)

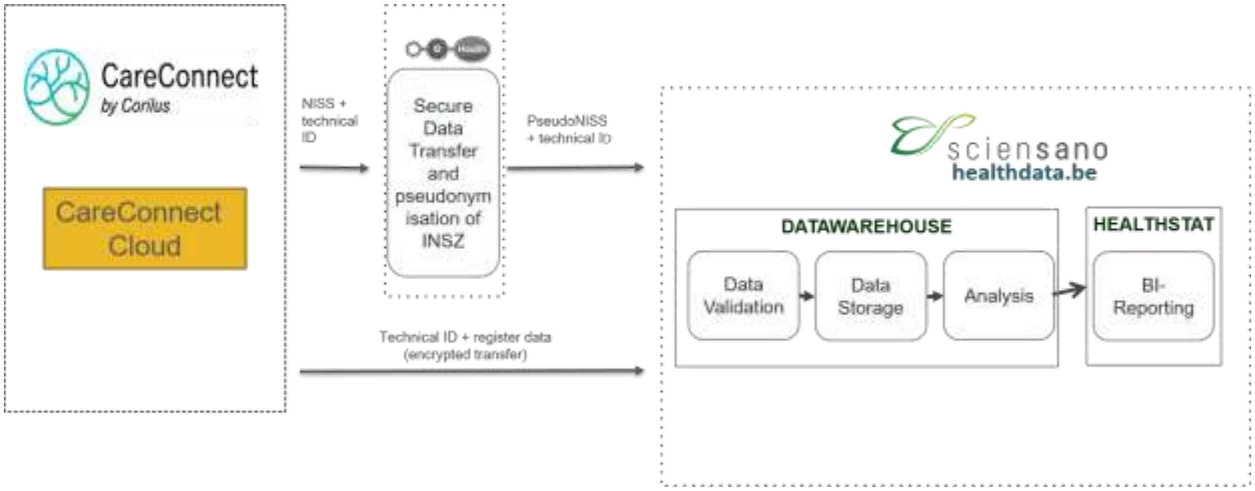

6.1. De huisartsen, die deelnemen aan INTEGO, maken gebruik van het elektronisch medisch dossier van CareConnect (Corilus). Datacollectie zal gebeuren door CSV-extracts vanuit de CareConnect cloud database richting het Sciensano healthdata.be-platform, hierbij rekening

[1] Het Comité herinnert eraan dat deze bijlage deel uitmaakt van deze beraadslaging. Er mag geen enkele wijziging worden aangebracht zonder de machtiging van de afdeling gezondheid van het Comité.

houdend met het feit dat er enkel data zullen worden geëxporteerd uit de CareConnect Cloud voor de artsen die deelnemen aan Intego en voor de patiënten die geen bezwaar hebben geuit. Er gebeurt een opsplitsing van de mededeling van persoonsgegevens door CareConnect aan het Healthdata.be-platform in, enerzijds, een afzonderlijke mededeling van de identificatiecode voor patiënten, en anderzijds een afzonderlijke mededeling van de registervariabelen.

6.2. De afzonderlijke mededeling van de identificatiecode voor patiënten (NISS), vergezeld van een door CareConnect gecreëerde technische registratiecode, wordt uitgevoerd middels de TTP-dienst van het eHealth-platform. Daarbij wordt door de verzender de technische registratiecode geëncrypteerd, de identificatiecode voor patiënten niet. Hierdoor pseudonimiseert de TTP-dienst van het eHealth-platform enkel de identificatiecode voor patiënten.

6.3. De afzonderlijke mededeling van de registervariabelen, vergezeld van de technische registratiecode (dezelfde als de technische code voor de identificatiecode voor patiënten), verloopt rechtstreeks (webservice) en geëncrypteerd van de verzender naar het healthdata.be-platform.

6.4. Het healthdata.be-platform decrypteert na ontvangst beide afzonderlijke mededelingen en consolideert deze op basis van de technische registratiecode. Na consolidatie en de technische kwaliteitscontrole, wordt de technische registratiecode onmiddellijk en definitief van de healthdata.be-infrastructuur verwijderd. Van deze technische processen zal het healthdata.be-platform een logging bijhouden.

6.5. Datavalidatie, databewaring en data-analyse: Op basis van de aangeleverde data voeren de onderzoekers hun werkzaamheden uit (onderzoek en opstellen van rapporten die ter beschikking worden gesteld van het publiek en specifieke doelgroepen). Zij krijgen hierbij enkel toegang tot de gegevens van hun toegewezen register (INTEGO).

6.6. Via healthstat.be kunnen op een geaggregeerd niveau wetenschappelijke rapporten, diagrammen en figuren van het INTEGO-register gedeeld worden met de buitenwereld. Dit gebeurt via een beveiligde webtoepassing.

6.7. De wijze waarop het INTEGO-register gebruik zou maken van data uit het Rijksregister wordt schematisch voorgesteld in figuur 2 en hieronder stapsgewijs beschreven. Deze wijze werd ter goedkeuring voorgelegd aan de dienst Toegang Rijksregister (FOD Binnenlandse Zaken)[2]:

- Wanneer een registratie voor het register ontvangen wordt voor een gecodeerd (gepseudonimiseerd) rijksregisternummer (INSZ of SSIN), dan wordt er aan de hand van het gecodeerde INSZ de eHealth webservice IdentifyEncodedPerson het rijksregister aangesproken. Deze webservice levert voor dit gecodeerde INSZ de overeenkomstige demografische gegevens uit het rijksregister. Voor INTEGO betreft het sterftedatum.

[2] FOD Binnenlandse Zaken, Algemene Directie Instellingen en Bevolking, beslissing nr. 020/2021 van 30 maart 2021 met betrekking tot de aanvraag die uitgaat van het Academisch Centrum Huisartsgeneeskunde KU Leuven, met het oog op het bekomen van toegang tot informatiegegevens van het Rijksregister en op het gebruiken van het Rijksregisternummer, met als doel het opnemen van de mortaliteitsstatus in het Intego-register

• Het oproepen van de IdentifyEncodedPerson webservice zorgt ook voor het oproepen van de ManageInscription webservice, die nodig is om in een 2e fase de updates te verkrijgen.

• Demografische gegevens die healthdata.be van het Rijksregister ontvangt, worden in een aparte database bijgehouden en gedistribueerd op basis van de machtiging van de data collectie (in dit geval het INTEGO register).

Figuur 2: Gebruik van de eHealth ConsultRR dienst door het healthdata.be-platform

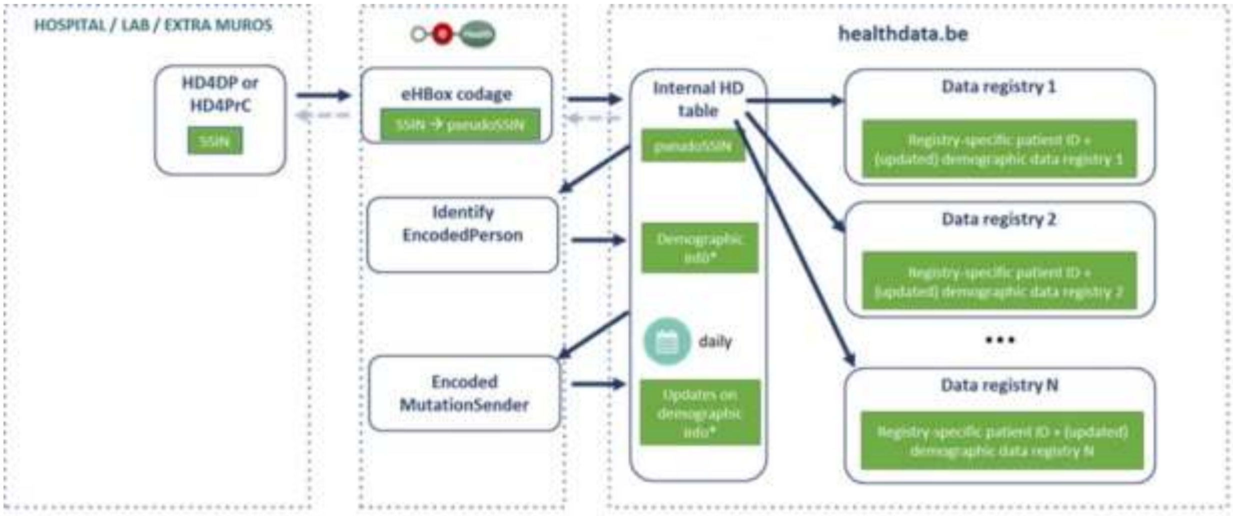

6.8. De hoger omschreven werkwijze zou dus volledig in lijn liggen met beraadslaging nr. 15/009 van het Informatieveiligheidscomité en te allen tijde waarborgen dat healthdata.be enkel gepseudonimiseerde INSZ-nummers blijft ontvangen, en dat de registermedewerkers enkel toegang hebben tot de parameters die proportioneel geacht worden door het Informatieveiligheidscomité.

<u>Deelname arts:</u>
Een arts binnen de praktijk kan aangeven, via het configuratiescherm van CareConnect, dat hij/zij wil deelnemen aan de Intego export. Daarbij geeft hij ook de inschrijvingsdatum mee dat hij/zij instapt in het project en vanaf wanneer hij zijn/haar patiënten zal informeren.

<u>Herinnering aan periodieke export</u>:
Zeven dagen voor de effectieve Intego-export zal elke deelnemende arts een e-mail notificatie ontvangen dat de export zal plaatsvinden. Tot het moment van export heeft de arts de tijd om zich uit te schrijven en zo geen export te laten plaatsvinden.

<u>Deelname patiënt</u>:

De export zelf zal enkel patiënten exporteren die na de inschrijfdatum een contact hebben gehad met de arts, zodat iedere patiënt de mogelijkheid heeft om de Intego-export te weigeren.

Een mogelijke weigering voor een individuele patiënt kan door de arts geregistreerd worden in het EMD op niveau van de patiënt.

7. Het eHealth-platform treedt op als intermediaire organisatie (Trusted Third Party). Een terugkoppeling/decodering is noodzakelijk om bevestigingsberichten (acknowledgements) te kunnen terugsturen vanuit healthdata naar de verzender (in dit geval CareConnect) om aldus op te kunnen volgen dat alle registraties correct aankomen bij healthdata.

8. Een small cell risk analyse zal door een arts van Sciensano[3] worden verricht.

9. Voor het beheer van de INTEGO-gegevensbank werden – op vraag van de Commissie voor de Bescherming van de Persoonlijke Levenssfeer[4] – twee adviesraden opgericht: een wetenschappelijk comité en een ethisch comité. Het wetenschappelijk comité is samengesteld uit vertegenwoordigers van het INTEGO-project, academici (artsen) en een externe arts. Het ziet toe op de kwaliteit van de verzamelde gegevens, de verwerking en de analyses. Het ethisch comité is samengesteld uit vertegenwoordigers van het INTEGO-project, academici (niet artsen) en een externe arts. Het ethisch comité ziet toe op de toepassing van de regels voor de bescherming van de persoonlijke levenssfeer en de ethische toepassingen van de verwerking van de gegevens. Bovendien wordt voor individuele projecten in voorkomend geval het advies van het medisch-ethisch comité van de faculteit geneeskunde van de KULeuven gevraagd.

10. De persoonsgegevens worden meegedeeld aan volgende instantie(s):

Instanties die toegang zullen krijgen tot gepseudonimiseerde, niet-geaggregeerde data:

• Academisch Centrum Huisartsgeneeskunde (ACHG- KULeuven)
• Onderzoekers werkzaam op het Intego-project via interuniversitaire samenwerking

Instanties die toegang zullen krijgen tot gepseudonimiseerde, geaggregeerde data (rapporten):

• De medewerkers in de registrerende huisartsencentra zullen toegang krijgen tot feedbackrapporten, waarin de gegevens van hun centrum zullen worden vergeleken met gemiddelde waarden van de andere centra. Deze rapporten kunnen geraadpleegd worden via het private luik van healthstat.be, de beveiligde rapporteringsomgeving van healthdata.be waarbij gebruikers zich door middel van eHealth Identity & Authorization Management dienen te authentificeren.
• Er zal de mogelijkheid zijn om rapporten op basis van geaggregeerde data ter beschikking te stellen van het brede publiek. Toegang tot deze rapporten zal verlopen via het publieke luik van healthstat.be.
• Het RIZIV, de federale en regionale ministeries van volksgezondheid.

---

[3] De identiteit van die arts werd aan het Comité overgemaakt.
[4] De modaliteiten van de gegevensverwerking werden in overleg met de Commissie voor de bescherming van de persoonlijke levenssfeer in 2007 uitgewerkt.

10.1. De gepseudonimiseerde persoonsgegevens van de INTEGO-gegevensbank kunnen voor het doeleinde van wetenschappelijk onderzoek ter beschikking worden gesteld aan:
- medewerkers van het ACHG die specifiek (contractueel) verbonden zijn aan het INTEGO-project;
- andere medewerkers van het ACHG;
- onderzoekers die niet verbonden zijn aan het ACHG.

10.2. Onderzoekers die niet verbonden zijn aan het ACHG en onderzoekers die wel verbonden zijn aan het ACHG maar niet aan het INTEGO-project, dienen een specifieke aanvraag tot toegang tot de INTEGO-databank in te dienen, waarna een overeenkomst wordt opgesteld waarin volgende aspecten worden opgenomen: de identificatie van de verantwoordelijke van de gegevensverwerking, de onderzoekers en hun respectieve functies, het doel van het onderzoek, de benodigde gegevens, de duur van het onderzoek met vermelding van de datum waarop de conversietabel wordt vernietigd (cfr. infra) en de vermelding dat de onderzoekers de privacywetgeving moeten respecteren. Deze overeenkomsten worden in beide adviesraden besproken. De aanvrager vermeldt dat deze ter beschikking van het Comité worden gehouden. Deze categorie van onderzoekers ontvangt in principe uitsluitend geaggregeerde gegevens. Indien het doeleinde van het onderzoek in kwestie effectief individuele gepseudonimiseerde persoonsgegevens zou vereisen, wordt slechts een selectie van de beschikbare gepseudonimiseerde persoonsgegevens ter beschikking gesteld op het datawarehouse van healthdata. Indien niet geaggregeerde gepseudonimiseerde persoonsgegevens aan deze categorie van onderzoekers worden meegedeeld dan worden de gecodeerde patiëntennummers voorafgaandelijk een derde maal[5] gecodeerd door het healthdata.be platform. De codes verschillen voor elke externe onderzoeksvraag.

10.3. De onderzoekers van het ACHG die specifiek verbonden zijn aan het INTEGO-project[6] kunnen toegang tot de gegevens vragen in het kader van het epidemiologisch onderzoek van het INTEGO-project. Deze personen overleggen regelmatig en kunnen verkennende, voorbereidende analyses doen op basis van bijvoorbeeld literatuur. Indien dit leidt tot een formele analyse van de gepseudonimiseerde persoonsgegevens uit de INTEGO-gegevensbank wordt dit meegedeeld aan de beide adviesraden. De resultaten van het epidemiologisch onderzoek worden op regelmatige wijze gepubliceerd en verspreid.

11. De meegedeelde persoonsgegevens zullen in gepseudonimiseerde vorm worden bewaard op het healthdata.be platform gedurende een periode van 30 jaar vanaf het overlijden van de betrokken patiënt.

II. BEVOEGDHEID

12. Ingevolge artikel 42, § 2, 3°, van de wet van 13 december 2006 houdende diverse bepalingen betreffende gezondheid vereist iedere mededeling van persoonsgegevens die de gezondheid betreffen, behoudens voorziene uitzonderingen, een principiële beraadslaging van de kamer sociale zekerheid en gezondheid van het Informatieveiligheidscomité.

---

[5] Na de eerste codering door het softwareprogramma en de tweede codering door de *trusted third party*.
[6] Het betreft thans drie personen waarvan het Comité de coördinaten mocht ontvangen.

13. De mededeling van gepseudonimiseerde persoonsgegevens die de gezondheid betreffen door huisartsen aan het ACHG van de KULeuven met het oog op de samenstelling van een register voor onderzoeksdoeleinden valt niet onder één van voormelde uitzonderingen, en vereist bijgevolg een beraadslaging van het Comité.

III. BEHANDELING VAN DE AANVRAAG

A. TOELAATBAARHEID

14. De verwerking van persoonsgegevens die de gezondheid betreffen is in principe verboden.[7]

15. Dit verbod geldt echter niet, zoals in casu het geval is, wanneer de verwerking noodzakelijk is met het oog op (…) wetenschappelijk (…) onderzoek (…) overeenkomstig artikel 89, lid 1, op grond van Unierecht of lidstatelijk recht, waarbij de evenredigheid met het nagestreefde doel wordt gewaarborgd, de wezenlijke inhoud van het recht op bescherming van persoonsgegevens wordt geëerbiedigd en passende en specifieke maatregelen worden getroffen ter bescherming van de grondrechten en de belangen van de betrokkene[8]. Dit is ook het geval wanneer de verwerking noodzakelijk is om redenen van algemeen belang op het gebied van de volksgezondheid, zoals bescherming tegen ernstige grensoverschrijdende gevaren voor de gezondheid (…), op grond van Unierecht of lidstatelijk recht waarin passende en specifieke maatregelen zijn opgenomen ter bescherming van de rechten en vrijheden van de betrokkene, met name van het beroepsgeheim[9].

16. Immers, de wet van 12 augustus 1911 tot toekenning van rechtspersoonlijkheid aan de hogescholen te Brussel en Leuven stelt dat de KU Leuven een instelling is met rechtspersoonlijkheid. Het verrichten van wetenschappelijk onderzoek is een taak die uitdrukkelijk is toegekend aan de Universiteiten in Vlaanderen door artikel II.18 van de Codex Hoger Onderwijs.

17. In het licht van het voorgaande is het Comité van oordeel dat er een toelaatbare grond bestaat voor de beoogde verwerking van gecodeerde persoonsgegevens die de gezondheid betreffen.

B. FINALITEIT

18. Persoonsgegevens dienen voor welbepaalde, uitdrukkelijk omschreven en gerechtvaardigde doeleinden te worden verkregen.

19. Het oorspronkelijke doeleinde van de gegevensverwerking was de samenstelling van een databank met gepseudonimiseerde gezondheidsgegevens om epidemiologisch en ander wetenschappelijk onderzoek mogelijk te maken. De KULeuven als zelfstandige universiteit heeft overeenkomstig haar statuten onder andere specifieke wetenschappelijke

---

[7] Artikel 9, §1 van de AVG.
[8] Artikel 9, §2, j) van de AVG.
[9] Artikel 9, §2, i) van de AVG.

onderzoeksopdrachten te vervullen. De oprichting van het INTEGO-register binnen het healthdata.be platform voldoet aan primaire en secondaire doelstellingen.

De primaire doelstellingen van Intego zijn het verzamelen en onderzoeken van epidemiologische gegevens omtrent ziekten zoals ze gediagnosticeerd worden in de huisartsenpraktijken. Het verzamelen en onderzoeken van socio-demografische gegevens van patiënten die in contact komen met hun huisarts.

De secundaire doelstelling is het onderzoeken van longitudinale gegevens om trends in de verzamelde epidemiologische gegevens mogelijk te maken, hetgeen tot het opstellen van nieuwe onderzoekshypotheses kan leiden.

20. Gelet op het voorgaande stelt het Comité dan ook vast dat de beoogde verwerking een welbepaald, uitdrukkelijk omschreven en gerechtvaardigd doeleinde heeft.

21. Overeenkomstig de AVG mogen persoonsgegevens niet verder worden verwerkt op een wijze die, rekening houdend met alle relevante factoren, met name met de redelijke verwachtingen van de betrokkene en met de toepasselijke wettelijke en reglementaire bepalingen, onverenigbaar is met de doeleinden waarvoor de gegevens oorspronkelijk werden verzameld.

C. PROPORTIONALITEIT

22. Persoonsgegevens dienen toereikend, terzake dienend en niet overmatig te zijn, uitgaande van de doeleinden waarvoor zij worden verkregen of waarvoor zij verder worden verwerkt.[10]

23. Het Comité stelt vast dat de betrokken personen de patiënten zijn van deelnemende huisartsen aan het project Intego die zich niet verzet hebben tegen de verwerking van hun persoonsgegevens in het kader van dit register. De gevraagde gegevens en de rechtvaardiging van de noodzaak ervan worden toegelicht in de bijlage bij die aangifte. Het Comité verklaart kennis te hebben genomen van deze gegevens en van de rechtvaardigingen door de aanvrager. Hierbij herinnert het Comité eraan dat iedere wijziging van deze gegevenslijst en van de rechtvaardigingen ervan door het Comité moet worden goedgekeurd.

24. Het Comité stelt vast dat de aanvrager een beslissing van de Minister van Binnenlandse Zaken heeft verkregen met betrekking tot de toegang tot bepaalde gegevens van het Rijksregister (datum van overlijden) en het gebruik van het rijksregisternummer.[11] Motivatie van de doeleinden voor deze toegang:

---

[10] Artikel 5 van de AVG.

[11] FOD Binnenlandse Zaken, Algemene Directie Instellingen en Bevolking, beslissing nr. 020/2021 van 30 maart 2021 met betrekking tot de aanvraag die uitgaat van het Academisch Centrum Huisartsgeneeskunde KU Leuven, met het oog op het bekomen van toegang tot informatiegegevens van het Rijksregister en op het gebruiken van het Rijksregisternummer, met als doel het opnemen van de mortaliteitsstatus in het Intego-register

- Accurate gegevens over mortaliteitsstatus kunnen gebruikt worden voor het opstellen van predictiemodellen inzake mortaliteit en morbiditeit voor diverse gezondheidsproblemen.
- Daarenboven kan de koppeling van eerstelijnsgegevens van het Intego-register met het gegeven inzake datum van overlijden uit het Rijksregister belangrijke trendgegevens bieden.

25. Het Comité stelt vast dat de beslissing van de Minister van Binnenlandse Zaken verleend wordt voor een periode van tien jaar. Bijgevolg herinnert het Comité eraan dat de koppeling van de gezondheidsgegevens van de patiënten met de gegevens van het rijksregister slechts toegelaten is voor de geldigheidsduur van de voormelde beslissing nr. 020/2021 van 30 maart 2021.

26. Het proportionaliteitsprincipe veronderstelt dat de verwerking in principe verricht wordt aan de hand van anonieme gegevens. Indien het doeleinde echter niet verwezenlijkt kan worden aan de hand van anonieme gegevens, kunnen gepseudonimiseerde persoonsgegevens worden verwerkt. Gelet op de noodzaak om zeer gedetailleerde analyses uit te voeren op basis van deze gegevens, heeft de aanvrager behoefte aan toegang tot gepseudonimiseerde gegevens om deze analyses te kunnen uitvoeren aangezien ze niet kunnen worden verricht aan de hand van anonieme gegevens. Dit doeleinde rechtvaardigt aldus de verwerking van gepseudonimiseerde persoonsgegevens.

27. Het Comité beschouwt de persoonsgegevens die aan de aanvrager zouden worden meegedeeld inderdaad als gepseudonimiseerde gegevens, vermits het identificatienummer dat voor de patiënt gebruikt wordt door het eHealth-platform gecodeerd wordt.

28. Wat de mededeling van gepseudonimiseerde persoonsgegevens door ACHG aan onderzoekers die niet verbonden zijn aan het INTEGO-project betreft, stelt het Comité vast dat er in wordt voorzien dat de finaliteit en de proportionaliteit van de gegevensverwerking door de ontvangers worden beoordeeld door een wetenschappelijk comité en door een ethisch comité, waarin zowel artsen als juristen betrokken zijn. Het Comité neemt eveneens akte dat de overeenkomsten die met de ontvangers worden opgesteld, ter beschikking worden gesteld voor toezicht.

29. Het Comité stelt vast dat een “small cell risk” analyse werd uitgevoerd door P-95.

30. Overeenkomstig artikel 5 van de AVG, mogen persoonsgegevens in een vorm die het mogelijk maakt de betrokkenen te identificeren, niet langer worden bewaard dan voor de verwezenlijking van de doeleinden waarvoor zij worden verkregen of verder worden verwerkt, noodzakelijk is. De aanvrager voorziet erin dat de gepseudonimiseerde persoonsgegevens in het Intego-register in gepseudonimiseerde vorm zullen worden bewaard gedurende een periode van 30 jaar na het overlijden van de betrokken patiënt. Na deze termijn mogen de gegevens uitsluitend in anonieme vorm worden bewaard, dit wil zeggen in een vorm die niet toelaat om de gegevens in verband te brengen met een geïdentificeerde of identificeerbare persoon.

31. Het Comité benadrukt dat de resultaten van de verwerking voor wetenschappelijke of statistische doeleinden niet in een vorm mogen worden bekendgemaakt die de identificatie van de betrokkene mogelijk maakt.

32. Het Comité acht de persoonsgegevens toereikend, terzake dienend en niet overmatig, uitgaande van de doeleinden waarvoor zij worden verkregen of waarvoor zij verder worden verwerkt.

D. TRANSPARANTIE

33. Overeenkomstig artikel 14 van de AVG verstrekt de verwerkingsverantwoordelijke de betrokkene de nodige informatie wanneer persoonsgegevens niet van de betrokkene zijn verkregen. Deze bepaling geldt echter niet, onder meer, wanneer het verstrekken van die informatie onmogelijk blijkt of onevenredig veel inspanning zou vergen. In dergelijke gevallen neemt de verwerkingsverantwoordelijke passende maatregelen om de rechten, de vrijheden en de gerechtvaardigde belangen van de betrokkene te beschermen, waaronder het openbaar maken van de informatie.

34. De aanvrager voorziet in een notificatie omtrent de verwerking van de gegevens ter attentie van de betrokkene via een affiche in de wachtzaal en de mogelijkheid om bijkomende informatie te vragen aan de huisarts tijdens de raadpleging. Het Comité stelt vast dat de informatie ook beschikbaar is op de website www.intego.be.

35. Het Comité herinnert eraan dat de informatie die beschikbaar is op de website alsook de affiche in de wachtzaal van de deelnemende huisarts regelmatig geactualiseerd dienen te worden.

36. Het Comité is aldus van oordeel dat er voldoende transparantie is omtrent de beoogde verwerking.

E. VEILIGHEIDSMAATREGELEN

37. Krachtens artikel 5 van de AVG moeten persoonsgegevens, door het nemen van passende technische of organisatorische maatregelen, op een dusdanige manier worden verwerkt dat een passende beveiliging ervan gewaarborgd is, en dat zij onder meer beschermd zijn tegen ongeoorloofde of onrechtmatige verwerking en tegen onopzettelijk verlies, vernietiging of beschadiging (integriteit en vertrouwelijkheid) Deze maatregelen moeten een passend beveiligingsniveau verzekeren, rekening houdend enerzijds met de stand van de techniek ter zake en de kosten voor het toepassen van de maatregelen en anderzijds met de aard van de te beveiligen gegevens en de potentiële risico's.

38. De verwerking met het oog op archivering in het algemeen belang, wetenschappelijk of historisch onderzoek of statistische doeleinden is onderworpen aan passende waarborgen in overeenstemming met de AVG voor de rechten en vrijheden van de betrokkene. Die waarborgen zorgen ervoor dat er technische en organisatorische maatregelen zijn getroffen om de inachtneming van het beginsel van minimale gegevensverwerking te garanderen. Deze maatregelen kunnen pseudonimisering omvatten, mits aldus die doeleinden in kwestie

kunnen worden verwezenlijkt. Wanneer die doeleinden kunnen worden verwezenlijkt door verdere verwerking die de identificatie van betrokkenen niet of niet langer toelaat, moeten zij aldus worden verwezenlijkt[12].

39. De verwerking van persoonsgegevens die de gezondheid betreffen moet gebeuren onder het toezicht en de verantwoordelijkheid van beroepsbeoefenaar in de gezondheidszorg[13]. Hoewel dit strikt genomen niet wordt vereist, verdient het volgens het Comité de voorkeur dat dergelijke gegevens worden verwerkt onder de verantwoordelijkheid van een geneesheer[14]. Het Comité mocht effectief de identiteit van de betrokken artsen van het Academisch Centrum Huisartsengeneeskunde van de KULeuven en van Sciensano ontvangen. Het Comité herinnert er aan dat de beroepsbeoefenaar in de gezondheidszorg en zijn aangestelden of gemachtigden bij de verwerking van persoonsgegevens tot geheimhouding verplicht zijn.

40. Overeenkomstig artikel 5 van de AVG moet de verantwoordelijke voor de verwerking moet de gepaste technische en organisatorische maatregelen treffen die nodig zijn voor de bescherming van de persoonsgegevens tegen toevallige of ongeoorloofde vernietiging, tegen toevallig verlies, evenals tegen de wijziging van of de toegang tot, en iedere andere niet toegelaten verwerking van persoonsgegevens.

41. De inzameling, de verwerking en de terbeschikkingstelling van de gecodeerde persoonsgegevens verlopen in overeenstemming met de voorwaarden vastgelegd in beraadslaging nr. 15/009 van 17 februari 2015, laatst gewijzigd op 3 maart 2020, betreffende de generieke methode voor de uitwisseling van gepseudonimiseerde persoonsgegevens die de gezondheid betreffen in het kader van healthdata.be en healthstat.be.

42. Om de vertrouwelijkheid en de veiligheid van de gegevensverwerking te garanderen, moet iedere instelling die persoonsgegevens bewaart, verwerkt of meedeelt afhankelijk van de context en de aard van de persoonsgegevens maatregelen nemen in de volgende elf actiedomeinen die betrekking hebben op de informatieveiligheid: veiligheidsbeleid; aanstelling van een functionaris voor gegevensbescherming; organisatorische en menselijke aspecten van de veiligheid (vertrouwelijkheidsverbintenis van het personeel, regelmatige informatieverstrekking en opleidingen ten behoeve van het personeel inzake bescherming van de privacy en veiligheidsregels); fysieke veiligheid en veiligheid van de omgeving; netwerkbeveiliging; logische toegangs- en netwerkbeveiliging; loggings, opsporing en analyse van de toegangen; toezicht, nazicht en onderhoud; systeem van beheer van de veiligheidsincidenten en de continuïteit (backup-systemen, fault tolerance-systemen, …); naleving en documentatie. Het Comité neemt akte van het feit dat de aanvrager bevestigt effectief te voorzien in alle vereiste veiligheidsmaatregelen, inclusief de aanstelling van een functionaris voor gegevensbescherming.

---

[12]Art. 89, §1, van de AVG
[13] Artikel 5 van de AVG.
[14] Beraadslaging nr. 07/034 van 4 september 2007.

43. Het Comité herinnert eraan dat krachtens artikel 9 van de wet van 30 juli 2018 *betreffende de bescherming van natuurlijke personen met betrekking tot de verwerking van persoonsgegevens* de verwerkingsverantwoordelijke de volgende bijkomende maatregelen neemt bij de verwerking van genetische, biometrische of gezondheidsgegevens:

1° hij of, in voorkomend geval, de verwerker wijst de categorieën van personen die toegang hebben tot de persoonsgegevens aan waarbij hun hoedanigheid ten opzichte van de verwerking van de betrokken gegevens nauwkeurig wordt omschreven;

2° hij of, in voorkomend geval, de verwerker houdt de lijst van de aldus aangewezen categorieën van personen ter beschikking van de bevoegde toezichthoudende autoriteit;

3° hij zorgt ervoor dat de aangewezen personen door een wettelijke of statutaire verplichting, of door een evenwaardige contractuele bepaling ertoe gehouden zijn het vertrouwelijk karakter van de betrokken gegevens in acht te nemen.

44. Het Comité herinnert er eveneens aan dat in het kader van de verwerking voor wetenschappelijke doeleinden de verwerkingsverantwoordelijke de bepalingen van titel 4 van de wet van 30 juli 2018 *betreffende de bescherming van natuurlijke personen met betrekking tot de verwerking van persoonsgegevens* moet naleven.

45. Het Comité wijst expliciet op de bepalingen van Titel 6. Sancties van de wet van 30 juli 2018 *betreffende de bescherming van natuurlijke personen met betrekking tot de verwerking van persoonsgegevens*, waarbij voorzien wordt in strenge administratieve sancties en strafsancties in hoofde van de verwerkingsverantwoordelijke en de verwerkers in geval van overtreding van de voorwaarden die bepaald zijn in de AVG en de voormelde wet van 30 juli 2018.

Om deze redenen, besluit

de kamer sociale zekerheid en gezondheid van het informatieveiligheidscomité,

gelet op de beslissing van FOD Binnenlandse Zaken nr. 016/2020 van 21 maart 2020 betreffende de aanvraag die uitgaat van het Academisch Centrum Huisartsgeneeskunde van de KU Leuven, met het oog op het bekomen van toegang tot informatiegegevens van het Rijksregister en met het oog op het gebruik van het Rijksregisternummer, in het kader van de COVID-19 pandemie en de beraadslaging nr 20/080 van 7 maart 2020 betreffende de toegang tot de Kruispuntbankregisters,

rekening houdend met beraadslaging nr. 15/009 van 17 februari 2015, laatst gewijzigd op 3 maart 2020, betreffende de generieke methode voor de uitwisseling van gepseudonimiseerde persoonsgegevens die de gezondheid betreffen in het kader van healthdata.be en healthstat.be,

dat de mededeling van persoonsgegevens, zoals beschreven in deze beraadslaging, is toegestaan mits wordt voldaan aan de vastgestelde maatregelen ter waarborging van de gegevensbescherming, in het bijzonder de maatregelen op het vlak van doelbinding, minimale gegevensverwerking, opslagbeperking en informatieveiligheid

Bart VIAENE
Voorzitter

De zetel van de kamer sociale zekerheid en gezondheid van het informatieveiligheidscomité is gevestigd in de kantoren van de Kruispuntbank van de Sociale Zekerheid, op volgend adres : Willebroekkaai 38 – 1000 Brussel.

Bijlage

Opsomming van de persoonsgegevens die worden meegedeeld en de rechtvaardiging van het noodzakelijk karakter

**9.1. Gegevens met betrekking tot socio-demografische gegevens**	
Beschrijving	INSZ (rijksregister- of bisnummer) van de patiënt. Volgens de standaardaanpak van healthdata.be wordt het INSZ tweemaal gecodeerd: een niet-register-specifieke codering door eHealth (eHealthbox batch codage), en een tweede register-specifieke codering door healthdata.
Reden	Eenduidige patiëntcodering om juiste statistieken te garanderen.
Beschrijving	Geboortejaar en -maand, geslacht, overlijdensstatus (ja/nee) en desgevallend overlijdensdatum, nationaliteit, burgerlijke staat, beroep, CG1 (verhoogde tegemoetkoming), postcode
Reden	De vitale status (incl. overlijdensdatum) wordt toegevoegd om een breder beeld te krijgen op de demografische gegevens. Nationaliteit wordt toegevoegd omdat de prevalentie en presentatie van ziekte kan verschillen naargelang de ethnische achtergrond van de patiënt (bv diabetes, hartfalen,…). Burgerlijke staat, beroep en CG1 wordt toegevoegd om de sociaal economische status van de patiënten mee te kunnen nemen in de analyses. Beroep is bovendien belangrijk in het onderzoeken van de relatie tussen beroep en morbiditeit.<br>De postcode wordt opgevraagd voor het achteraf mogelijk maken van analyse van het geografisch voorkomen van bepaalde aandoeningen.<br>Gegevens over mortaliteitsstatus worden gebruikt worden voor het opstellen van predictiemodellen inzake mortaliteit en morbiditeit.
Beschrijving	Behandelende arts, identificatie praktijk
Reden	De identificatie van de arts aan de hand van RIZIV-nummer laat toe om gepersonaliseerde feedbackrapporten te kunnen terugsturen. Dit is een basisfunctionaliteit die door healthdata.be aangeboden wordt. Enkel de 3 laatste cijfers (specialisme) zullen beschikbaar zijn voor analyse.<br>Het identificatienummer van de praktijk wordt gevraagd om analyses per praktijk mogelijk te maken. In vele analyses wordt de identificatie van de praktijk als random effect meegenomen.
**9.2. Gegevens met betrekking tot diagnoses**	
Beschrijving	• Zorgelement en startdatum zorgelement<br>• Status (actief/passief) en datum wijziging status<br>• Evaluatie en datum evaluatie<br>• Aanmeldingsklacht en datum aanmeldingsklacht

Reden	Het ontwikkelen van een longitudinale gegevensbank van ziekten die gegevens kan verschaffen over de incidentie en de prevalentie van ziekten in Vlaanderen.
**9.3. Gegevens met betrekking tot medicatie**	
Beschrijving	• Identificatiecode van de medicatie en typering code<br>• Start- en stopdatum<br>• Status (chronisch, acuut, éénmalig of indien nodig)<br>• Posologie
Reden	Het ontwikkelen van een longitudinale gegevensbank van voorgeschreven medicatie die gegevens kan verschaffen over de evolutie van medicatie voorgeschreven door huisartsen in Vlaanderen.
**9.4. Gegevens met betrekking tot labo**	
Beschrijving	Medidoc code + datum labo aanvraag
Reden	Het ontwikkelen van een longitudinale gegevensbank van aangevraagde labotesten die gegevens kan verschaffen over de evolutie van aanvragen van labotesten door huisartsen in Vlaanderen.
Beschrijving	Resultaat, eenheid en referentiewaarde
Reden	Het resultaat van de labotesten is van belang omdat deze vaak gebruikt worden als 'proxy' voor de aanwezigheid van ziekte, of om de ernst van de ziekte in te schatten.
**9.5. Gegevens met betrekking tot vaccinaties**	
Beschrijving	• ATC code en CNK code (VOS, INN of VMP)<br>• Datum van toediening
Reden	Het ontwikkelen van een longitudinale gegevensbank van uitgevoerde vaccinaties die gegevens kan verschaffen over de evolutie vaccinatie door huisartsen in Vlaanderen.
**9.6. Gegevens met betrekking tot behandelingen**	
Beschrijving	• Titel behandeling<br>• Datum behandeling
Reden	De behandeling is van belang om de gevolgen van een aandoening te onderzoeken en de ernst van de aandoening te kunnen inschatten.
**9.7. Gegevens met betrekking tot parameters**	

**9.7.1. Gegevens met betrekking tot vitale en fysieke parameters**	
Beschrijving	• Lengte en datum lengte<br>• Gewicht en datum gewicht<br>• BMI en datum BMI<br>• Diastolische en systolische bloeddruk en datum diastolische en systolische bloeddruk<br>• Hartslag en datum hartslag<br>• Ritme en datum ritme<br>• Temperatuur en datum temperatuur<br>• Buikomtrek en datum buikomtrek<br>• Zuurstofsaturatie en datum zuurstofsaturatie<br>• Lichaamsbeweging en datum lichaamsbeweging<br>• Monofilament test en datum<br>• Perifere pulsaties en datum
Reden	Het onderzoeken van de evolutie van vitale en fysieke parameters is van belang in relatie tot de aanwezigheid van ziekte en medicatievoorschriften en het ontwikkelen van risicopredictiemodellen.
**9.7.2. Gegevens met betrekking tot rookstatus en alcoholgebruik**	
Beschrijving	Rookstatus en datum rookstatus
Reden	Het onderzoeken van de evolutie van rookgedrag in Vlaanderen. Bovendien is het onderzoeken van de leefstijl, zoals roken, van belang in relatie tot het ontstaan van ziekte.
Beschrijving	Alcoholgebruik en datum alcoholgebruik
Reden	Het onderzoeken van de evolutie van alcoholgebruik in Vlaanderen. Bovendien is het onderzoeken van de leefstijl, zoals alcoholgebruik, van belang in relatie tot het ontstaan van ziekte.
**9.8. Gegevens met betrekking tot specifieke parametersets**	
**9.8.1. Gegevens met betrekking tot longfunctie**	
Beschrijving	• Forced Expiratory Volume 1 (FEV1) + datum<br>• GOLD classificatie (spirometrie) + datum<br>• Forced Vital Capacity (FVC) + datum<br>• Tiffeneau Index + datum

	• Peak-flow + datum
Reden	Het onderzoeken van de evolutie van de longfunctie en de ernst van luchtwegaandoeningen.
**9.8.2. Gegevens met betrekking tot nierfunctie**	
Beschrijving	• Stadium nierinsufficiëntie + datum<br>• Eiwit dosage urine + datum<br>• Microalbumine urine + datum
Reden	Het onderzoeken van de evolutie van de nierfunctie en de ernst van de nierinsufficiëntie.
**9.8.3. Gegevens met betrekking tot hartfunctie**	
Beschrijving	• CHA2DS2-VASc-score (score voor risico op voorkamerfibrillatie) + datum<br>• QRS complex (ECG) besluit + datum<br>• ST segment (ECG) besluit + datum<br>• PQ-interval (ECG) + datum<br>• ECG uitgevoerd? + datum<br>• Kortademigheid NYHA classifcatie (graad 0-4) + datum NYHA
Reden	Het onderzoeken van de evolutie van de hartfunctie en de ernst van de hartaandoening.
**9.9. Gegevens met betrekking tot allergieën**	
Beschrijving	Allergeen + datum allergie
Reden	Het ontwikkelen van een longitudinale gegevensbank van allergieën die gegevens kan verschaffen over de incidentie en de prevalentie van allergieën in Vlaanderen.
**9.10. Gegevens met betrekking tot medicatie intolerantie**	
Beschrijving	• Verpakking<br>• datum medicatie-intolerantie
Reden	Belangrijk in het begrijpen wanneer bepaalde medicatie niet werd voorgeschreven. Op deze manier kan de data rond medicatievoorschriften beter geïnterpreteerd worden.
**9.11. Gegevens met betrekking tot familiegeschiedenis**	
Beschrijving	• diagnose

	• relatie
Reden	Belangrijk in het identificeren van risicopatiënten en het ontwikkelen van risico-predictiemodellen.
**9.12. Gegevens met betrekking tot zorgplannen**	
Beschrijving	• titel<br>○ voortraject diabetes<br>○ zorgtraject chronische nierinsufficiëntie<br>○ zorgtraject diabetes type II<br>• start- en einddatum
Reden	Het onderzoeken van ziektemanagement en zicht krijgen op de kwaliteit van de geleverde zorg.
**9.13. Gegevens met betrekking tot afwezigheidsattest**	
Beschrijving	Start- en einddatum
Reden	Het beter kunnen inschatten van de impact van de aanwezigheid van ziekte.
**9.14. Gegevens met betrekking tot beeldvorming**	
Beschrijving	Datum aanvraag medische beeldvorming
Reden	Enerzijds zicht krijgen op de evolutie van aanvragen voor medische beeldvorming, en anderzijds zicht krijgen op het diagnostisch proces bij het vermoeden van ziekte.
**9.15. Gegevens met betrekking tot kinesitherapievoorschrift**	
Beschrijving	Datum voorschrift kinesitherapie
Reden	De behandeling, zoals kinesitherapie, is van belang om de gevolgen van een aandoening te onderzoeken en de ernst van de aandoening te kunnen inschatten.
