# Supplementary material for: Benchmarks for low back pain in general practice in Flanders: electronic audit of INTEGO
Source: BMC Prim Care. 2024 Dec 20;25:431. doi: 10.1186/s12875-024-02644-6 (PMC11660715; doi:10.1186/s12875-024-02644-6)
Supplement: Supplementary file 2 — Supplementary Material 2. [file 12875_2024_2644_MOESM2_ESM.pdf]

GROEP BIOMEDISCHE WETENSCHAPPEN
HERESTRAAT 49, O&N II – BUS 700
BE-3000 LEUVEN

English version below.

Ons kenmerk: MP018709
Uw kenmerk: Masterproef studie: Low back pain in general practice: design and evaluation of an automated clinical audit: Imaging, farmacotherapy and physical therapy
Leuven, 21-12-2021

DEFINITIEF GUNSTIG ADVIES

Geachte Bert Vaes
Geachte Charlotte De Clercq, Rico Paridaens

De Ethische Commissie Onderzoek UZ/KU Leuven verleent hierbij een gunstig advies aan de voorgestelde studie, zoals ze werd beschreven in het protocol. De Commissie is van oordeel dat er vanuit ethisch standpunt geen bezwaren zijn bij de voorgestelde studie. De studie werd goedgekeurd op 21-12-2021

De Onderwijs-Begeleidings-Commissie voor Medische Ethiek (OBC) heeft in delegatie van het EC Onderzoek UZ/KU Leuven het vermeld protocol onderzocht en besproken op haar vergadering.

(Goedkeuring) This is a retrospective observational study ai

Aangezien het om een Masterproefproject binnen de Groep Biomedische Wetenschappen van de KU Leuven gaat, werd de aanvraag tot de Ethische Commissie Onderzoek UZ/KU Leuven ingediend via de Onderwijs-Begeleidings-Commissie.

De Commissie heeft geen bezwaar tegen het project mits vertrouwelijke behandeling van de gegevens en naleving van de Belgische wetgeving omtrent privacy. De Ethische Commissie Onderzoek UZ KU Leuven wenst de hoofdonderzoeker/promotor van de studie te wijzen op zijn/haar verantwoordelijkheid betreft de privacy van de persoons-/patiënt-gegevens bij contact met levende personen, patiënten en/of inzage in het elektronisch medisch dossier, inclusief de correcte implementatie hiervan door medewerkers en studenten.

Dit gunstig advies betreft de indiening van 01-12-2021 en wordt gegeven voor de duur van de Masterproef van de betrokken student(en). Elke wijziging aan het protocol doet dit gunstig advies vervallen. U dient in dat geval tijdig een amendement voor advies voor te leggen aan de commissie die eerder uw dossier goedkeurde.

De Commissie bevestigt dat ze werkt in overeenstemming met de ICH-GCP principes (International Conference on Harmonization Guidelines on Good Clinical Practice), met de meest recente versie van de Verklaring van Helsinki en met de van toepassing zijnde wetten en regelgeving.

De Commissie bevestigt dat in geval van belangenconflict, de betrokken leden niet deelnemen aan de besluitvorming omtrent de studie.

Met vriendelijke groet,

Prof. dr. Minne Casteels
Voorzitter

Ethische Commissie Onderzoek UZ/KU Leuven

Prof. dr. Pascal Borry
Voorzitter
Onderwijs-BegeleidingsCommissie voor Medische Ethiek KU Leuvenv

---

Aandachtspunten (indien van toepassing)

De Ethische Commissie UZ KU Leuven wenst de hoofdonderzoeker/promotor van de studie te wijzen op zijn/haar verantwoordelijkheid betreft de privacy van de persoons-/patiëntgegevens bij contact met de patiënt en/of inzage in het elektronisch medisch dossier, inclusief de correcte implementatie hiervan door medewerkers en studenten. Het EC verwijst naar de richtlijnen van ICH/GCP hierover op de website, en benadrukt dat een GCP-opleiding van elke hoofdonderzoeker verwacht wordt voor studies die vallen onder de Wet op Medische Experimenten. Het EC verwijst tevens naar de Belgische wetgeving (Wet van 8/12/1992 ter bescherming van de persoonlijke levenssfeer en Wet van 22/8/2002 betreffende de rechten van de patiënt), en de Algemene Verordening Gegevensbescherming (van toepassing op 25 mei 2018).

Bij gebruik van elektronisch medische gegevens is een formeel akkoord van de dienstraad vereist. De opdrachtgever is verantwoordelijk voor de conformiteit van de anderstalige documenten met de Nederlandstalige documenten.

Onderzoek op embryo's in vitro valt onder de wet van 11 mei 2003. Voor dergelijk onderzoek is er naast een positief advies van het EC Onderzoek UZ/KU Leuven ook een goedkeuring van de Federale Commissie voor medisch en wetenschappelijk onderzoek op embryo's in vitro noodzakelijk vooraleer dit onderzoeksproject kan doorgaan.

Vooraleer u dierexperimenten mag opstarten is een goedkeuring vereist van de Ethische Commissie Dierproeven (ECD). U dient zich bijgevolg aan te melden bij deze commissie voor het onderdeel met dierproeven en hun adviezen te volgen. Gelieve te noteren dat u het onderzoek pas mag starten nadat u een definitief gunstig advies van deze commissie heeft verkregen.

Onderzoek op humane kadavers vereist een goedkeuring van het Ethisch Comité voor Zorg en Begeleiding. U dient zich bijgevolg aan te melden bij deze commissie en hun adviezen te volgen. Gelieve te noteren dat u het onderzoek pas mag starten nadat u een definitief gunstig advies van deze commissie heeft verkregen

Dit gunstig advies van de Commissie houdt niet in dat zij de verantwoordelijkheid voor de geplande studie op zich neemt. U blijft hiervoor dus zelf verantwoordelijk.
Bovendien dient u, als betrokken hoofdonderzoeker, erover te waken dat de resultaten van dit onderzoek, correct worden weergegeven in publicaties, rapporten voor de overheid enz.

U wordt eraan herinnerd dat bij klinische studies iedere door u waargenomen ernstige verwikkeling onmiddellijk zowel aan de opdrachtgever (desgevallend de producent) als aan het EC Onderzoek UZ/KU Leuven moet worden gemeld, ook al is het oorzakelijke verband met de studie onduidelijk.

Our reference: MP018709
Your reference: Master's thesis study: Low back pain in general practice: design and evaluation of an automated clinical audit: Imaging, farmacotherapy and physical therapy
Leuven, 21-12-2021

DEFINITIVE FAVOURABLE ADVICE

Dear Bert Vaes
Dear Charlotte De Clercq, Rico Paridaens

The Research Ethics Committee UZ/KU Leuven hereby grants favourable advice to the proposed study, as it was described in the protocol. The Commission is of the opinion that from an ethical standpoint there are no objections to the proposed study. The study was approved on 21-12-2021

De Onderwijs-Begeleidings-Commissie voor Medische Ethiek (OBC) heeft in delegatie van het EC Onderzoek UZ/KU Leuven het vermeld protocol onderzocht en besproken op haar vergadering.

(Goedkeuring) This is a retrospective observational study ai

Given that the study takes the form of a Master's thesis within the Group Biomedical Science, the application to the Research Ethics Committee UZ/KU Leuven was submitted to the Educational-Support Committee.

The Committee has no objection to the project on the basis of confidential treatment of the data and compliance with the Belgian legislation concerning privacy. The Research Ethics Committee UZ/KU Leuven wishes for the head researcher / promoter of the study to be aware of their responsibilities with regard to the privacy of personal / patient details, when in contact with living persons, patients, and / or contained within electronic medical records, including the correct implementation by employees and students.

This favorable advice concerns the submission of 01-12-2021 and is given for the duration of the Master's thesis of the student(s) concerned. Any amendment to the protocol will invalidate this favorable advice. In that case, you must submit an amendment for advice to the committee that previously approved your file.

The Committee confirms that they work in accordance with the ICH-GCP principles (International Conference on Harmonisation Guidelines on Good Clinical Practice), with the most recent verison of the Helsinki Declaration, and with applicable laws and regulations.

The Committee confirms that in case of a conflict of interest, the members concerned have not participated in decision regarding the study.

Kind regards,

Prof. dr. Minne Casteels
President / Chairperson
Research Ethics Committee UZ/KU Leuven

Prof. dr. Pascal Borry
President / Chairperson
Education-Support Committee for Medical Ethics KU Leuven

Points of Interests (if applicable)

The Research Ethics Committee UZ/KU Leuven wishes for the head researcher / promoter of the study to be aware of their responsiblities with regard to the privacy of personal / patient details, when in contact with living persons, patients, and / or contained within electronic medical records, including the correct implementation by employees and students. The Ethics Committee refers to the guidelines of the ICH/GCP here on the website, and emphasises that GCP-training is expected from every head-researcher for studies that come under the Law on Medical Experimentation. The Ethics Committee refers at the same time to the Belgian legislation (Law from 8/12/1992 for the protection of personal privacy and Law from 22/8/2002 regarding the rights of the patient), and the General Data Protection Regulation (which comes into effect on May 25th 2018).

For use of electronic medical data, a formal agreement from the clinical board is required. The study promotor is responsible for the conformity of foreign language documents with the Dutch language version.

In vitro research on embryos comes under the Law of 11 May 2003. For such research, besides positive approval from the Ethics Committee, approval by the Federal Committee for Medical and Scientific Research on Embryos In Vitro is also necessary before a project can commence.

Before you may commence animal experimentation, approval is required from the Animal Ethics Committee. You must register with this committee for the animal testing component and follow their advice. Please note that you may start the investigation only after you have obtained a definitive favorable opinion from this committee.

Research on human cadavers requires approval of the Care and Support Ethics Committee. You must therefore register with this committee and follow their advice. Please note that you may start the investigation only after you have obtained a definitive favorable opinion from this committee.

This favorable opinion from the Commission does not mean that it takes responsibility for the planned study. You remain responsible for this yourself. In addition, as the principal investigator, you must ensure that the results of this research are correctly displayed in publications, reports for the government, etc.

You are reminded that any serious complication you observe must be reported immediately to both the study promotor (if applicable, the producer) and the Ethics Committee, even if the causal link with the study is unclear.
