# Supplementary material for: Benchmarks for low back pain in general practice in Flanders: electronic audit of INTEGO
Source: BMC Prim Care. 2024 Dec 20;25:431. doi: 10.1186/s12875-024-02644-6 (PMC11660715; doi:10.1186/s12875-024-02644-6)
Supplement: Supplementary file 3 — Supplementary Material 3. [file 12875_2024_2644_MOESM3_ESM.pdf]

GROEP BIOMEDISCHE WETENSCHAPPEN
Academisch Centrum voor
Huisartsgeneeskunde
HERESTRAAT 49, O&N II – BUS 700
BE-3000 LEUVEN

MASTERPAPER PROTOCOL: RETROSPECTIVE STUDY

1. APPLICANTS

*This application can be prepared in **Dutch** or in **English**. The blue text boxes with explanation can be deleted when submitting the application. See the website for examples and text suggestion for the various sections. (https://med.kuleuven.be/nl/obc/Documents_en)*

***Every resubmission or amendment** should be done in **'track changes' or in 'highlight'** in order to identify the changes. The file name must also be changed to 'resubmission_MP###_version#' or 'amendment_MP##_version#'. The MP-number can be found via SCONE (overview master thesis), the version number is up to you: starting at 1 and increasing by 1 each time you are asked to make new changes.*

**Protocol - full title**	Low back pain in general practice: design and evaluation of an automated clinical audit: Imaging, farmacotherapy and physical therapy
**Version 1: Date submission**	Date:Click here to enter a date.
**Version 2: Date resubmission**	(to add in case of resubmission) Date:Click here to enter a date.
**Version 3: Date third submission**	(to add in case of third submission) Date:Click here to enter a date.
**Principal Investigator – Promoter**	Name: Bert Vaes, MD, PhD
	Telephone: +32 (0)16 337 468
	E-mail: bert.vaes@kuleuven.be
**Co-Investigator – Co-Promoter**	Name: Bert Aertgeerts, MD PhD
	Telephone: +32 (0)16 37 72 78
	Email: bert.aertgeerts@kuleuven.be
**Sub-investigator – Student(s)**	Name: Rico Paridaens, MD
	Student number: 0901134
	Telephone: +32 (0)470 83 39 09
	Email: rico.paridaens@kuleuven.be
	Name: Charlotte De Clercq, MD
	Student number: r0380440
	Telephone: +32 (0)496 38 94 44
	Email: csdeclercq@gmail.com

2. BACKGROUND AND RATIONALE

Oliveira et al. [1] define non-specific low back pain (LBP) as “low back pain not attributable to a known cause” [1]. 90-95 % of LBP cases are non-specific [1,2]. These cases of LBP generally don’t lead to absence from work and for those it does, three quarters of them return to work within 4 weeks. However a small part develop long-lasting LBP which leads to long-term absence from work [2,3]. Additionally LBP is the leading cause of years lived with disability [1]

An estimated 60 to 90 % of the Western population will experience at least one episode of low back pain (LBP) during their lifetime, of which 30 % has a second episode within the year [4–6]. In Flanders low back pain is part of the top 10 diseases that general practitioners diagnose each year (incidence 43,7 per 1,000 patients in 2015) [7].

An evidence-based approach to the management of LBP may decrease the amount of imaging requested and reduce the amount of prescriptions for physiotherapy, opioids and nonsteroidal anti-inflammatory drugs (NSAID) while resulting in the same outcome [8]. In Belgium the KCE, *Federaal* k*enniscentrum voor de gezondheidszorg*, implemented new guidelines for the management of LBP in 2019 aiming to improve the quality of care for LBP [3]. The adherence to these guidelines, and more generally the quality of care of the management of low back pain, in Flanders is however unknown.

Commonly, audit and feedback (A&F) is used to promote the implementation of evidence based medicine. Ivers et al.[9] define audit and feedback (A&F) as: “providing a recipient with a summary of their performance over a specified period of time.” Giving practitioners feedback on their current practice may improve adherence to evidence based medicine by self-assessment or self-monitoring [9]. However, the results of A&F are variable ranging from a relatively large, positive effect on quality of care (one-quarter) to a negative or null effect (one-quarter) [9,10]. In medicine we generally speak of a clinical audit, which is a continuous “quality loop” aiming to highlight the discrepancies between actual practice and standard of care. This “quality loop” consists of: choosing a topic, setting criteria and a standard of care, collecting data followed by data analysis and change implementation and, finally, checking for improvement and maintaining it [11,12]. The evolution in electronic medical records the past years allows for the opportunity to do these audits electronically which allows general practitioners (GP) to do these audits more time-efficiently and also make these results reproducible. This allows comparison of the results between GPs [9].

Currently no electronic clinical audits are available in Belgium to evaluate the management of low back pain in general practice. In this master dissertation we will aim to create an electronic clinical audit for the management of low back pain and check the validity of this clinical audit for use in daily practice. The clinical audit “quality loop” will be used in this process.

3. STUDY OBJECTIVES

Development of an electronic clinical AUDIT for the evaluation of low back pain in general practice in Flanders and evaluate the validity of this AUDIT. In total 4 quality indicators will be implemented in the AUDIT. These quality indicators were defined by CEBAM, the Belgian Centrum for Evidence-Based Medicine (https://www.cebam.be/). These quality indicators are:

1. % of adults with low back pain where imaging is requested
2. % of adults with low back pain that get a prescription for opioids compared to the amount of adults with low back pain that get a prescription for non-steroidal anti-inflammatory drugs (NSAID)
3. % of adults with low back pain where revalidations is prescribed
4. Employment status of people with low back pain

4. RESEARCH METHOD

Present the methodologies and explain potential ethical implications of the planned methodologies.

Based upon the quality indicators defined earlier a protocol will be developed that allows data collection from the electronic medical record software CareConnect General Practitioner from Corilus using the Statistics module. However, we know that the Statistics module available in CareConnect has limitations. Therefore, this protocol will be further refined and custom programmed in CareConnect as a standardized electronic AUDIT to collect all information correctly. In a second step each included patient will be reviewed manually by one of the researchers using the same protocol to evaluate if the data collected by the electronical AUDIT is correct. All data will be collected by general practitioners in training who also work in the participating practice where the data is collected. No information on individual patients will be exported out of the EMD (for example date of birth, name, place of residence). Only aggregated data will be exported and reported for the participating practices. An ethical implication may be that this AUDIT will only be developed in CareConnect. However a general protocol will be developed, which can also be used by other EMD software developers to implement this clinical AUDIT. The performance of each GP practice will be compared to a benchmark in the literature. Since the population profile in private practices can influence the result further subgroup analysis will be done (age, gender…) to compensate for these differences in population.

5. DATA COLLECTION

Specify the source and method of the data to be collected / obtained and specify the time period that will be studied.
Note: As this concerns a retrospective study, the past will be examined using already rightfully collected data from files that are:
- medical files of the study participants

- other accessible data files/bases with collected personal data of the study participants, e.g. pharmaceutical files, vaccination data bases, RIZIV data bases, disease registers, …

The data will be collected in 2 general practitioner practices in Flanders, “Groepspraktijk Huisartsen Harelbeke” and “Huisartsenpraktijk Nieuwenhoven”. The data is collected retrospectively using consultation reports between 01/10/2020 and 30/09/2021. Only aggregated data will be reported for the participating practices. No data on individual patients will leave the GP practice. Data analysis using individual patient data will be performed within the GP practice.

Contact information:

- Groepspraktijk Huisartsen Harelbeke, Kortrijksesteenweg 28, 8530 Harelbeke, Dr. Stijn Torbeyns, groepspraktijk.harelbeke@skynet.be, 056 71 54 41
- Huisartsenpraktijk Nieuwenhoven: Nieuwenhovenlaan 4, 1600 Sint-Pieters-Leeuw, Dr. Siegfried Geens, mail@siegfriedgeens.be, 02/377.51.78

6. ANALYSIS

Describe the measures taken to avoid, or at least minimize, biases. Give the number of participants to be enrolled, together with the rationale for the sample size. Describe also methods for data analysis.

The two datasets (electronic and manual AUDIT) in each practice will be further analysed using MathWorks MATLAB R2018a. The table below lists all collected data and the statistical analyses applied to the data. A significance level of 0.05 will be used. Before each test the requirements for the test will be checked. All variables are unpaired. Since this project is a pilot project no specific minimal sample size is defined, however the sample size should suffice for the necessary statistical analysis since 2 private practices will be evaluated with an estimated number of active global medical record patients of close to 5000 patients each.

Indicator
Demographic information<br>• Gender<br>• Age<br>• Nationality
Medical background information<br>• Comorbidities<br>• Chronic medication

- Number of previous episodes of low back pain

Indicator 1: % of adults with low back pain where imaging is requested

- Imaging requested?
  Descriptive statistics
  Chi²-test with H_0: there is no difference between the amount of people that received imaging between the manual AUDIT and the electronic AUDIT population
  Two proportion Z-test with H_0: the amount of imaging requested in the manual AUDIT population (respectively electronic AUDIT population) is comparable to the benchmark found in literature.
- Type of imaging requested
  Descriptive statistics
  If Gaussian distribution criteria are met: unpaired parametric Student's t-test, otherwise Mann-Whitney U-test, with H_0: there is no difference between the type of imaging request between the manual AUDIT and the electronic AUDIT population
  Two proportion Z-test with H_0: the type of imaging requested in the manual AUDIT population (respectively electronic AUDIT population) is comparable to the benchmark found in literature.

Indicator 2: % of adults with low back pain that get a prescription for opioids compared to the amount of adults with low back pain that get a prescription for non-steroidal anti-inflammatory drugs (NSAID)

- Medication prescribed?
  Descriptive statistics
  Chi²-test with H_0: there is no difference between the amount of people that received a prescription for medication between the manual AUDIT and the electronic AUDIT population
  Two proportion Z-test with H_0: the frequency of medication prescribed in the manual AUDIT population (respectively electronic AUDIT population) is comparable to the benchmark found in literature.
- Which medication?
  Descriptive statistics
  If Gaussian distribution criteria are met: unpaired parametric Student's t-test, otherwise Mann-Whitney U-test, with H_0: there is no difference between the type of medication prescribed between the manual AUDIT and the electronic AUDIT population
  Two proportion Z-test with H_0: the amount of opioids (respectively NSAID) prescribed in the manual AUDIT population (respectively electronic AUDIT population) is comparable to the benchmark found in literature.

Indicator 3: % of adults with low back pain where physiotherapy is prescribed

- Revalidations prescribed?
  Descriptive statistics
  Chi²-test with H_0: there is no difference between the amount of people that received physiotherapy between the manual AUDIT and the electronic AUDIT population
  Two proportion Z-test with H_0: the frequency of the physiotherapy prescribed in the manual AUDIT population (respectively electronic AUDIT population) is comparable to the benchmark found in literature.
- Type of Revalidations: physical therapy vs group sessions in hospital
  Descriptive statistics
  If Gaussian distribution criteria are met: unpaired parametric Student's t-test, otherwise Mann-Whitney U-test, with H_0: there is no difference between the type of physiotherapy prescribed between the manual AUDIT and the electronic AUDIT population
  Two proportion Z-test with H_0: the amount of physiotherapy prescriptions in the manual AUDIT population (respectively electronic AUDIT population) is comparable to the benchmark found in literature.

Indicator 4: Employment status of people with low back pain

- Certificate of absence prescribed?
  Descriptive statistics
  Chi²-test with H_0: there is no difference between the amount of people that received a certificate of absence between the manual AUDIT and the electronic AUDIT population
  Two proportion Z-test with H_0: the frequency of incapacity prescribed in the manual AUDIT population (respectively electronic AUDIT population) is comparable to the benchmark found in literature.
- Duration of incapacity
  Descriptive statistics
  If Gaussian distribution criteria are met: unpaired parametric Student's t-test, otherwise Mann-Whitney U-test, with H_0: there is no difference between the duration of incapacity prescribed between the manual AUDIT and the electronic AUDIT population
  Two proportion Z-test with H_0: the duration of incapacity prescribed in the manual AUDIT population (respectively electronic AUDIT population) is comparable to the benchmark found in literature.

7. DATA HANDLING AND MANAGEMENT

7.1 Data storage and management

Explain which type of data you are using, how the privacy of the participants is being protected (e.g. coding or anonymization), how the data is safely stored, and what will happen to the data at the end of the project.

The data will be collected in 2 general practitioner private practices in Flanders. Only patients with an active global medical file registered to one of the GPs of the practice will be included. The data collected using the electronic AUDIT is anonymized automatically upon export out of CareConnect since no individual patient data is exported, only practice wide data is exported. The data collected manually will be collected by 2 medical doctors who are bound by medical privacy and who are the 2 primary researchers of this master dissertation. They will anonymize the data within the private practice so no data that can identify a patient (i.e. name, date of birth, national register numbers, …) will leave the electronic medical record (Careconnect). The anonymized data will be further analyzed using statistical software. The anonymized data will be kept for 2 years after analysis and deleted afterwards. Only the processed data will be used in this master thesis.

7.2 Declaration of Confidentiality and Careful Management of Information and Personal Data

In the framework of his / her Master's thesis, the student(s) will have access to all kinds of data, information, results and documents, including personal data which is not exclusively limited to patient data (the "Information"). In order to ensure the confidentiality of this Information and the privacy of those involved, within the framework of his / her Master's thesis the student(s) should always deal with the Information with the greatest care and discretion.

Data collected in the framework of a Master's thesis, in particular research related to patients and including the collection and analysis of personal data, requires the utmost of care and discretion. Therefore, at all times the student must observe complete confidentiality with respect to the Information he / she has collected during the course of his / her Master's thesis.

In performing this research, the student(s) commit(s) him/herself to the following confidentiality obligations:

- *He / she accepts, during and after the completion of the Master's thesis, the obligation to strictly observe the confidentiality of the Information he / she has collected and the activities to which he / she has participated, and regarding the patients, healthy volunteers and the staff members with whom he / she comes into contact;*
- *He / she will only process and collect data that is relevant and necessary for his / her Master's thesis;*
- *He / she will not share information with persons not directly involved within the framework of his / her research;*
- *He / she will take all necessary steps to protect the confidentiality of Information and the privacy of those involved;*
- *He / she will handle with care and responsibility the Information and the access granted to him/her to information systems and digital media.*

  All investigators shall treat all information and data relating to the study as confidential and shall not disclose such information to third parties or use such information for any purpose other than the performance of the study. The collection, processing and disclosure of personal data, such as participants' health and medical information, is subject to compliance with applicable personal data protection legislation.

8. APPROVAL

Hereby we confirm that data collection is performed with approval of the head of the respective unit(s) or department(s) where data collecting is taking place. The data will be collected in 2 private practices of general practitioners.

9. PUBLICATION POLICY

Publications will be coordinated by the Principal Investigator. Authorship to publications will be determined in accordance with the requirements published by the International Committee of Medical Journal Editors and in accordance with the requirements of the respective journal.

10. DIRECT ACCESS TO SOURCE DATA AND DOCUMENTS

The investigator(s) and the institution(s) will permit study-related monitoring, audits, ethical review and regulatory inspections (where appropriate) by providing direct access to source data.

11. REFERENCES

1. Oliveira CB, Maher CG, Pinto RZ, Traeger AC, Lin CWC, Chenot JF, et al. Clinical practice guidelines for the management of non-specific low back pain in primary care: an updated overview. European Spine Journal. 2018;27(11):2791–803.
2. Staal JB, Hendriks EJM, Heijmans M, Kiers H, Lutgers-Boomsma AM, Rutten G, et al. KNGF-richtlijn: Lage rugpijn (update klinimetrie 2017). Fysiopraxis. 2017;13.
3. KCE Report. Klinische richtlijn rond lage rugpijn en radiculaire pijn 2017. 2017;
4. De Groot F, Kolnaar B, Chavannes A. NHG-standaard aspecifieke lagerugpijn. Huisarts en Wetenschap. 2005;48(5):251.
5. Malmivaara A, Pohjolainen T, Hirvensalo E, Jousimaa J. Duodecim Richtlijn: Lage rugpijn. Duodecim Medical Publications Ltd. 2017.
6. Maher C, Underwood M, Buchbinder R. Non-specific low back pain. The Lancet. 2017 Feb;389(10070):736–47.
7. Truyers C, Goderis G, Dewitte H, Akker MV, Buntinx F. The Intego database: Background, methods and basic results of a Flemish general practice-based continuous morbidity registration project. BMC Medical Informatics and Decision Making. 2014;14(1):1–9.
8. McGuirk B, King W, Govind J, Lowry J, Bogduk N. Safety, Efficacy, and Cost Effectiveness of Evidence-Based Guidelines for the Management of Acute Low Back Pain in Primary Care: Spine. 2001 Dec;26(23):2615–22.
9. Ivers NM, Sales A, Colquhoun H, Michie S, Foy R, Francis JJ, et al. No more 'business as usual' with audit and feedback interventions: Towards an agenda for a reinvigorated intervention. Implementation Science. 2014;9(1):1–8.
10. Jamtvedt G, Young JM, Kristoffersen DT, O'Brien MA, Oxman AD. Audit and feedback: effects on professional practice and health care outcomes. Cochrane Database of Systematic Reviews. 2006;
11. Esposito P. Clinical audit, a valuable tool to improve quality of care: General methodology and applications in nephrology. World Journal of Nephrology. 2014;3(4):249.
12. Benjamin A. The competent novice. Audit: How to do it in practice. Bmj. 2008;336(7655):1241–5.
